# Seasonal variation of airborne fungal diversity and community structure in urban outdoor environments in Tianjin, China

**DOI:** 10.3389/fmicb.2022.1043224

**Published:** 2023-01-09

**Authors:** Yumna Nageen, Xiao Wang, Lorenzo Pecoraro

**Affiliations:** School of Pharmaceutical Science and Technology, Tianjin University, Tianjin, China

**Keywords:** airborne fungi, environmental factors, fungal diversity, concentration, and community structure, green and busy urban areas, outdoor environments, seasonal variation

## Abstract

Airborne fungi are ubiquitous in human living environments and may be a source of respiratory problems, allergies, and other health issues. A 12 months study was performed to investigate the diversity, concentration and community structure of culturable airborne fungi in different outdoor environments of Tianjin City, using an HAS-100B air sampler. A total of 1,015 fungal strains belonging to 175 species and 82 genera of Ascomycota 92.5%, Basidiomycota 7%, and Mucoromycota 0.3% were isolated and identified using morphological and molecular analysis. The most abundant fungal genera were *Alternaria* 35%, *Cladosporium* 18%, *Penicillium* 5.6%, *Talaromyces* 3.9%, *Didymella* 3%, and *Aspergillus* 2.8%, while the most frequently occurring species were *A. alternata* (24.7%), *C. cladosporioides* (11%), *A. tenuissima* (5.3%), *P. oxalicum* (4.53%), and *T. funiculosus* (2.66%). The fungal concentration ranged from 0 to 340 CFU/m^3^ during the whole study. Environmental factors, including temperature, relative humidity, wind speed, and air pressure exerted a varying effect on the presence and concentration of different fungal taxa. The four analyzed seasons showed significantly different airborne fungal communities, which were more strongly influenced by air temperature and relative humidity in spring and summer, whereas wind speed and air pressure had a stronger effect in autumn and winter. Fungal communities from green and busy sites did not show significant differences over the four analyzed seasons, which may be due to the effect of the surrounding environments characterized by high human activities on the air of the relatively small parks present in Tianjin. The present study provided valuable information on the seasonal dynamics and the environmental factors shaping the diversity and concentration of the analyzed outdoor airborne fungal communities, which can be of help for air quality monitoring, microbial contamination control, and health risk assessment in urban environments.

## 1. Introduction

Fungi are one of the most pervasive groups of living organisms in nature, playing vital roles in the environment as saprotrophs, symbionts or parasites (Cvetnić and Pepeljnjak, 1997; Anees-Hill et al., 2022; Martins, 2022). Approximately 25% of the global biomass is estimated to be constituted by fungi (Miller, 1992; Burge, 2001). Fungal spores are commonly found dispersed in the atmosphere, thus being named as airborne (Cvetnić and Pepeljnjak, 1997). The concentration of airborne fungi in different environments varies depending on a number of factors, including fungal substrates availability and human activity Miller, 1992; Sorenson, 1999; Kurup et al., 2000; Adhikari et al., 2004). Airborne fungi are considered to be linked to air pollution and implicated in negative health consequences on humans, animals, and plants (Bush and Portnoy, 2001; Shelton et al., 2002; Martinez-Bracero et al., 2022). Approximately, 150 allergenic fungal *taxa* have been identified to date (Simon-Nobbe et al., 2008). About 10% of the entire human world population is estimated to have fungal allergies, whereas 40% of the asthma patients are supposed to show fungal sensitivity (Burge, 2001). Almost 80 fungal genera have been reported to be associated with respiratory tract allergies (Latge and Paris, 1991; Horner et al., 1995; Hughes et al., 2022). Besides, more than a hundred fungal species have been found to be accountable for different human and animal diseases, while many others are responsible for plant diseases (Cvetnić and Pepeljnjak, 1997).

Many fungi are cause of pathological infestations in humans (Pei-Chih et al., 2000; Kasprzyk et al., 2021). Previous research studies have shown that airborne fungi are linked to various respiratory ailments (Pei-Chih et al., 2000; Patel et al., 2018; Odebode et al., 2020; Sio et al., 2021). These include some less infectious diseases like Aspergillosis (Anderson et al., 1996), hypersensitivity diseases, such as asthma and hypersensitivity pneumonitis (Cuijpers et al., 1995; Hu et al., 1997; Pashley and Wardlaw, 2021), and some toxicosis reactions, such as acute systemic toxicosis (Flannigan et al., 1991). The genera *Alternaria, Aspergillus, Cladosporium*, and *Penicillium* are the most common allergenic fungal taxa (Shelton et al., 2002; Adhikari et al., 2004; Fang et al., 2005; Abdel Hameed et al., 2009). *Alternaria* is considered one of the dominant sources of aeroallergens, being responsible for allergic rhinitis (Mokhtari Amirmajdi et al., 2011), and important risk factor for developing Asthma (Salo et al., 2006; Lehmann et al., 2016; Hayes et al., 2018; Hernandez-Ramirez et al., 2021; Sanchez et al., 2022). *Aspergillus* fungi are ubiquitous saprotrophs, with about 20 species known to cause human diseases (Dagenais and Keller, 2009). *Aspergillus fumigatus is the most abundant species in this fungal genus, causing about 90% of all invasive aspergillosis, while A. niger induces a variety of invasive lung illnesses* (Marr et al., 2002)*. Cladosporium* fungal species are mostly saprotrophic, and rarely associated with opportunistic infections in human and animals (Ma et al., 2013; Mercier et al., 2013; Sandoval-Denis et al., 2015; Batra et al., 2019). They have also been found to cause allergic rhinitis (Sellart-Altisent et al., 2007), deep or localized superficial lesions (Gugnani et al., 2000, 2006; Sang et al., 2012) and disseminated infections (Kantarcioglu et al., 2002; Kantarcioglu and de Hoog, 2004; Lalueza et al., 2011).

The concentration of airborne fungi varies across different months and seasons in a year depending on environmental factors such as temperature, relative humidity, wind speed, and air pressure (Lin and Li, 2000; Ponce et al., 2013). Air temperature and humidity are important factors to enhance the propagation of airborne fungi, as they sustain fungal growth and germination (Fang et al., 2005, 2019). Wind is also a significant factor influencing fungal communities, as it helps in the detachment of spores from fungal fruit bodies and conidiophores, and their dispersal and suspension in the air (Picco and Rodolfi, 2000;Giri et al., 2008; Pyrri and Kapsanaki-Gotsi, 2017). Atmospheric pressure enhances the suspension of fungal spores in the air by preventing their sedimentation, while helping their transportation and dispersal (Giri et al., 2008; Pyrri and Kapsanaki-Gotsi, 2017). Air pollution is a crucial factor influencing climatic conditions, thus possibly affecting, either directly or indirectly, the fungal diversity, distribution and concentration in the air (Pyrri and Kapsanaki-Gotsi, 2017).

Many research investigations have been carried out around the world aiming at finding the relationship between airborne fungi and plant, animal and human diseases, as well as the link between fungal presence and concentration in the air and spoilage of food (Rossi et al., 2005; Tournas and Katsoudas, 2005; Karmakar et al., 2020). The analysis of airborne fungal communities in different human living environments can be of crucial help for medical evaluation, health risk assessment and air quality monitoring (Shelton et al., 2002). Identifying the fungal species and their relative frequencies in a particular environment can aid in assessing the health risk posed by airborne fungi (Shelton et al., 2002). Various studies on outdoor airborne fungi have been conducted in Chinese cities, including Tianjin (Nageen et al., 2021), Beijing (Fang et al., 2005, 2008), Hangzhou (Fang et al., 2019), and Tainan (Pei-Chih et al., 2000), which contributed to enrich our basic knowledge about fungal presence and diversity in the atmosphere.

Tianjin, being the third largest municipality and the leading port of North China, is a city of great economic importance (Baruch Boxer, 2022). In a previous recent study, we have provided the first assessment of Tianjin outdoor airborne fungal diversity in different urban environments, using the open plate sampling method during a winter season (Nageen et al., 2021). The purpose of the present study was to comprehensively evaluate the concentration and distribution of airborne fungi in eight different outdoor environments of Tianjin City for a period of a whole year, in order to understand the effect of seasonal variation of the environment on the fungal community structure and dynamics. The present study aimed to provide detailed information on culturable airborne fungal diversity sampled using a volumetric air sampler, and to analyze the effect of different environmental factors on the studied fungal community. The results of this study may represent a reference for the prevention and control of microbial contamination in urban outdoor environments.

## 2. Materials and methods

### 2.1. Sampling sites

The study was carried out in Tianjin city, which is one of the biggest and economically most important cities in northern China. Tianjin is bordered by Hebei province and Beijing municipality, and bounded by the Bohai Gulf portion of the Yellow Sea to the east. It exhibits a continental climate, with temperature extremes ranging between −22.9 and 40.5°C (https://wikitravel.org/en/Tianjin, n.d.). It occupies an area of 11,946 square kilometers, including 15 districts and three counties, with a population of 13,794,450 in 2021 (World Population Review, n.d.).

**Figure 1:**
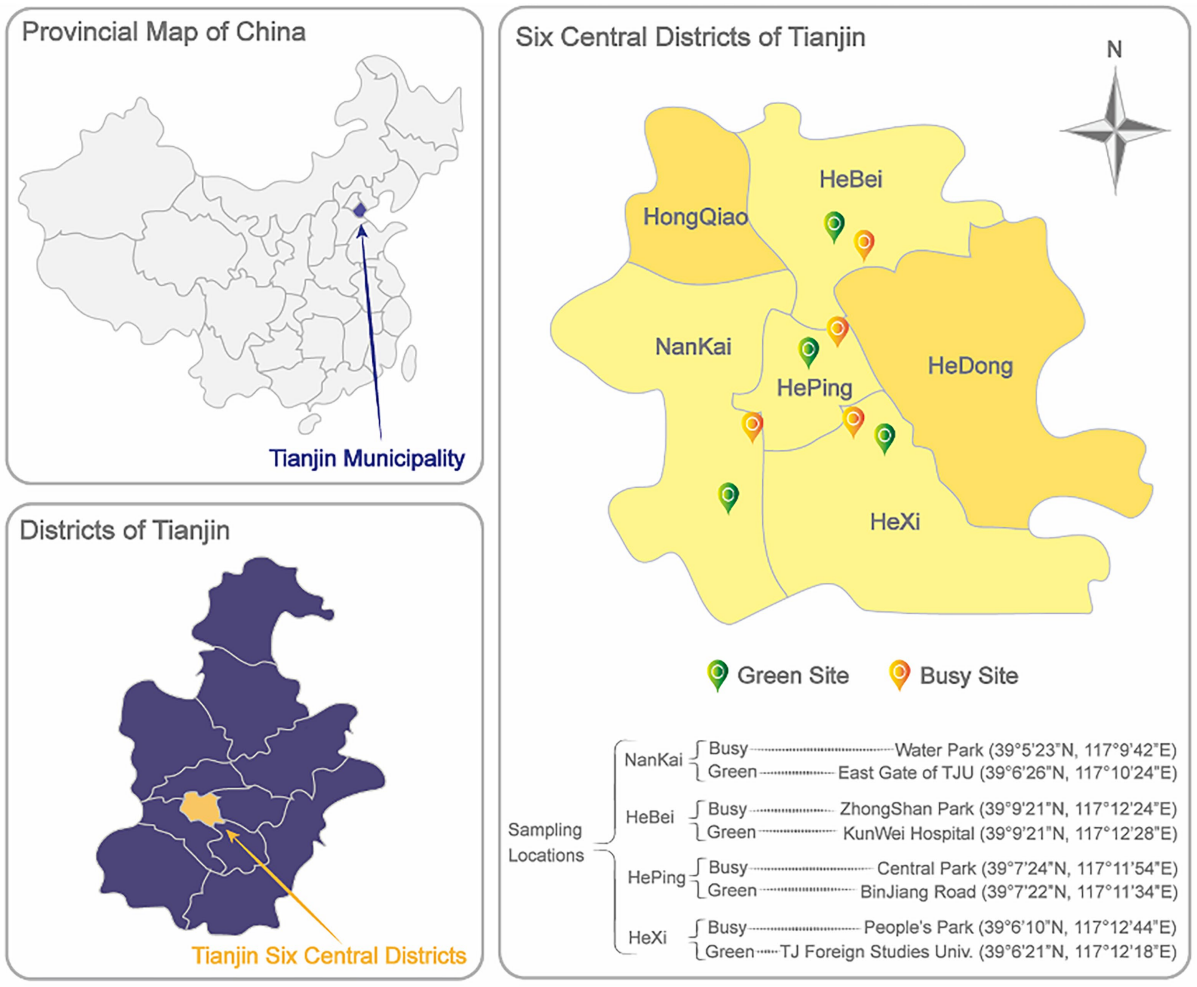
Schematic diagrams of Tianjin Municipality location in China, and distribution of Tianjin total and central districts. Detailed eight sampling locations and coordinates in NanKai, HeBei, HePing, and HeXi districts are marked in the diagram.

Air sampling was conducted at eight outdoor sites located in the 4 main urban districts across Tianjin city. For each district, 2 different sampling environments were selected, one constituted by a busy area with high traffic flow and human activities (busy site) and one represented by a green area in an urban park (green site) as shown in Figure 1.

### 2.2. Sampling and fungal isolation

Sampling was conducted once a month for a period of 1 year from April 2020 to March 2021, using the impaction method, with a sampler HAS-100 B (Hengao T&D, China), at each of the eight studied sites. The sampler, containing a petri dish of 9 cm diameter filled with Malt Extract Agar (MEA) medium amended with Ampicillin (100 mg/L) to prevent bacterial growth, was mounted on a support at 1.5 m above the ground level, and operated at a flow rate of 100 L/min and rotating dish speed of 0–4 rpm for 10 min at each location. Before each sampling, the sampler was swabbed using 70% ethanol to prevent cross-contamination. Every sampling was performed in the morning time, when the weather was sunny and stable. Environmental factors, including temperature and relative humidity, were recorded using a TES 1364 Humidity-Temperature Meter (Hengao T&D, China), while wind speed and air pressure data were retrieved from The Weather Company (n.d.).^1^ After sampling, the capsules were sealed with parafilm and transported back to the laboratory, where they were kept in an incubator for 7 days at 25°C, in the darkness. The plates were regularly observed every 24 h to detect fungal growth. Growing colonies were counted and reported as colony-forming units per cubic meter of air. CFU/m^3^ was calculated using the following formula (Fang et al., 2005):


$$\begin{array}{l} \left(\mathrm{Number of colonies}\times 1,000\right)\\ /\left(\mathrm{Sampling time}\times \mathrm{Velocity of}\;\mathrm{air}\;\mathrm{flow}\right) \end{array}$$

Fungal colonies from each plate were aseptically transferred in new 6.0 cm diameter plates for isolation. All the isolated strains were preserved and deposited in the LP Culture Collection (personal culture collection held in the laboratory of Prof. Lorenzo Pecoraro), at the School of Pharmaceutical Science and Technology, Tianjin University, Tianjin, China.

### 2.3. Fungal identification

Isolated fungal colonies were identified based on molecular and morphological analyses. For the molecular identification, fungal DNA was extracted using the cetyltrimethylammonium bromide method (Möller et al., 1992). The fungal rRNA genes internal transcribed spacer (ITS) region was amplified using universal primers ITS1 (5′-TCCGTAGGTGAACCTGCGG-3′) and ITS4 (5′-TCCTCCGCTTATTGATATGC-3′; White et al., 1990). The polymerase chain reactions were performed in a 50 μl total volume, consisting of 25.0 μl of 2× Rapid Taq Master Mix (Vazyme, China), 2 μl of both forward and reverse primers (10 μM), 2 μl (20 ng DNA) of DNA template and 19 μl of double distilled sterilized water. The amplification program consisted of initial denaturation at 95°C for 3 min, 35 cycles of 95°C for 15 s, annealing at 60°C for 15 s, extension at 72°C for 15 s, followed by a final elongation at 72°C for 5 min using a Mastercycler® thermal cycler (Eppendorf, Germany). The PCR products were analyzed using Gel electrophoresis in 1% agarose gel stained with Gel Red (TsingKe, China), using a reference 2000 bp ladder marker (TsingKe, China). The sequencing of PCR products was performed at Tsingke Biological Technology Company (Beijing, China). The sequences were analyzed using Basic Local Alignment Search Tool (BLAST) program of the National Center for Biotechnology Information, USA^2^ to determine the closest matches that enabled taxonomic identification. Fungal DNA sequences were submitted to GenBank under accessions ON711628-ON712642. Fungal morphology was characterized based on macroscopic and microscopic observations (Pecoraro et al., 2020; Wang and Pecoraro, 2021). Microscopic morphological traits, such as septate hyphae, phialides, hyphal coils, conidiophores, conidia, and conidial chains were examined under a Nikon ECLIPSE Ci-L microscope (Tokyo, Japan) for identification of isolates following the taxonomic keys for different taxa (Gilman, 1957; Lucas, 1981; De Hoog et al., 2000).

### 2.4. Statistical analysis

The data collected in this study were analyzed based on regression analysis and one way analysis of variance (ANOVA) using statistical package for social science (IBM SPSS Version 22.0), Microsoft excel 2010 and Multivariate Statistical Package (MSVP 3.22). Graphs were made using GraphPad Prism 8.3.0. The fungal diversity was analyzed based on the Shannon index while the differences in fungal concentrations under various environmental conditions (temperature, relative humidity, wind, and air pressure) were evaluated using Pearson Correlation Analysis. Krona chart to illustrate the taxonomic identification and relative abundance of fungi was made using Krona Tools.^3^

3D Principal Co-ordinates Analysis (3D PCoA) and Analysis of Similarity (ANOSIM) with 999 permutations were performed based on the Bray-Curtis distance in R package. Distance-based Redundancy Analysis (dbRDA) was performed to relate the environmental variables with the culturable fungal community based on Bray–Curtis distance matrice. The envfit function (999 permutations) in the vegan package of R was used to test the statistical significance of environmental factors. Kruskal-Wallis test was used to explore variations of abundant genera among four seasons. Adjusted value of *p* < 0.05 was considered statistically significant. *Post hoc* Dunn’s test was used to further compare the differences between multiple pairwise groups after Kruskal-Wallis test. Between-groups Venn diagram was plotted using R to identify unique and common fungal genera. Correlations between fungal genera and environmental parameters were evaluated based on Spearman’s correlation coefficients (*r*). The differences in fungal genera between busy and green sites in each season were assessed by Wilcoxon rank-sum test.

## 3. Results

### 3.1. Total fungal strains isolated

The total number of airborne fungal colonies collected in this study varied from different locations (Supplementary Figure 1A) and months (Supplementary Figure 1B). Concerning the analyzed sites, the highest number of strains was recorded at Hebei G (156), followed by Heping G (141), Nankai G (138), Heping B (131), and Hexi B (127), while a lower number of strains were isolated for Hebei B, Nankai B and Hexi G, which yielded 113, 107, and 102 fungal strains, respectively. Concerning months, the highest number of strains were recorded in May (188), followed by December (132), April (127), and October (116), while the lowest number of strains was recorded in the months of March (52), January (32), and February (16).

### 3.2. Airborne fungal community diversity, distribution, and concentration

**Figure 2:**
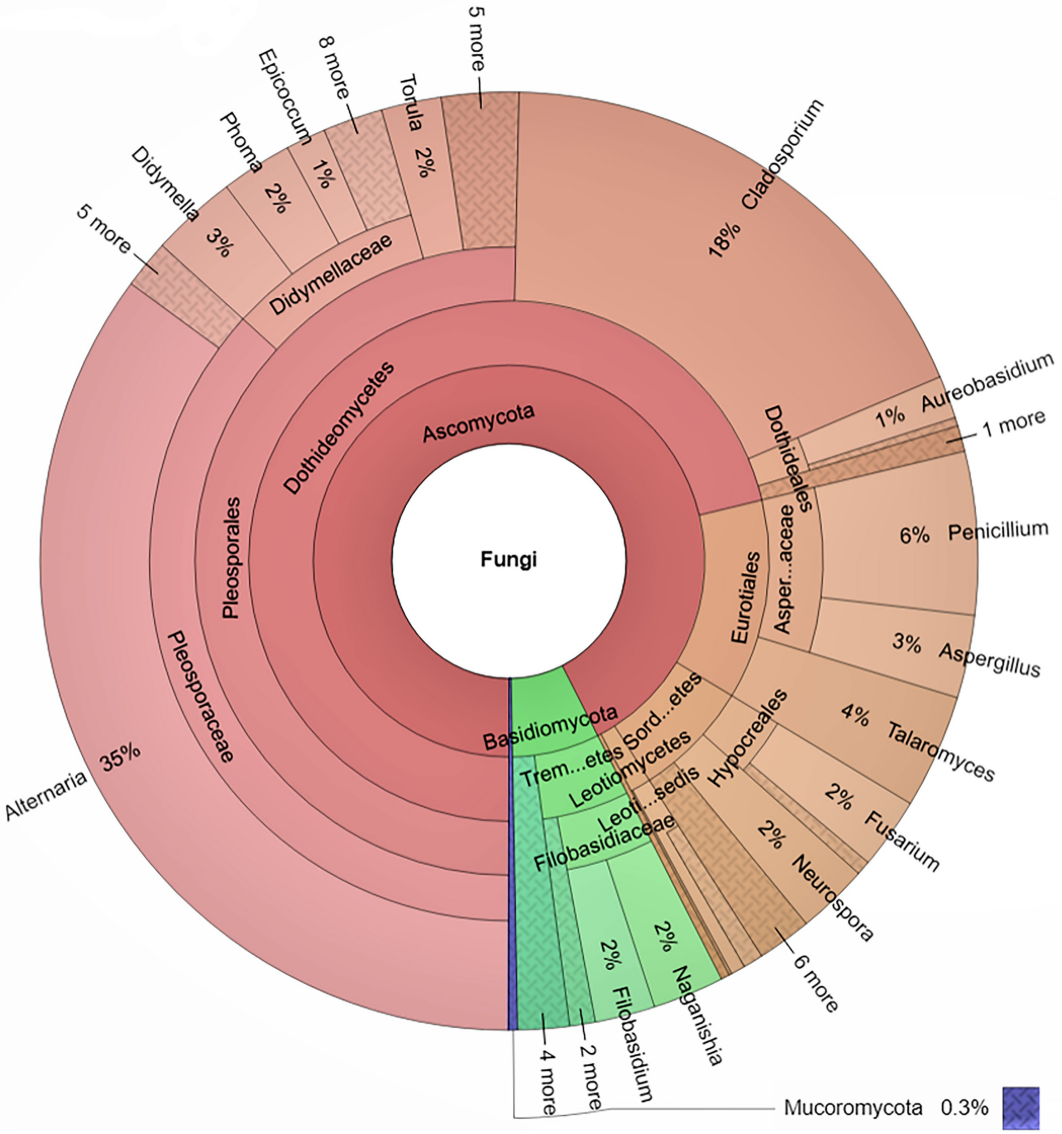
Krona Chart indicating the taxonomic identification and relative abundance of fungi isolated from Tianjin air.

**Figure 3:**
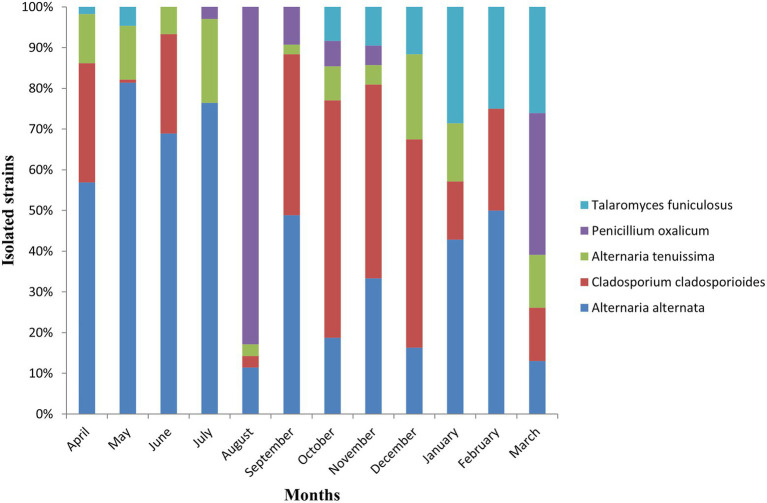
Strains of the dominant fungal species isolated each month.

A total of 1,015 fungal strains belonging to 3 phyla, 11 classes, 28 orders, 54 families, 82 Genera and 175 species were isolated during the whole one-year long study (Figure 2). The great majority of isolated strains (92.5%) belonged to Ascomycota, while Basidiomycota accounted for 7% of the fungal diversity, and Mucoromycota for 0.3%. *Alternaria* was the dominant genus, including 35% of total fungal strains, followed by *Cladosporium* 18%, *Penicillium* 5.6%, *Talaromyces* 3.9%, *Didymella* 3%, and *Aspergillus* 2.8%. These dominant fungal genera showed varying levels of abundance in different months (Supplementary Figure 2). At species level, *Alternaria alternata* (24.7%) was the most common fungus found throughout the year, showing particularly high relative abundance from April to July (Figure 3). *Cladosporium cladosporioides* (11%) was the second most abundant fungal species, showing a peak in relative abundance between October and December, followed by *Alternaria tenuissima* (5.3%), *Penicillium oxalicum* (4.53%) and *Talaromyces funiculosus* (2.66%). In particular, the presence of *Penicillium oxalicum* in Tianjin air environments was mostly concentrated in the month of August, whereas the highest relative abundance of *Talaromyces funiculosus* was recorded from January to March (Figure 3).

**Figure 4:**
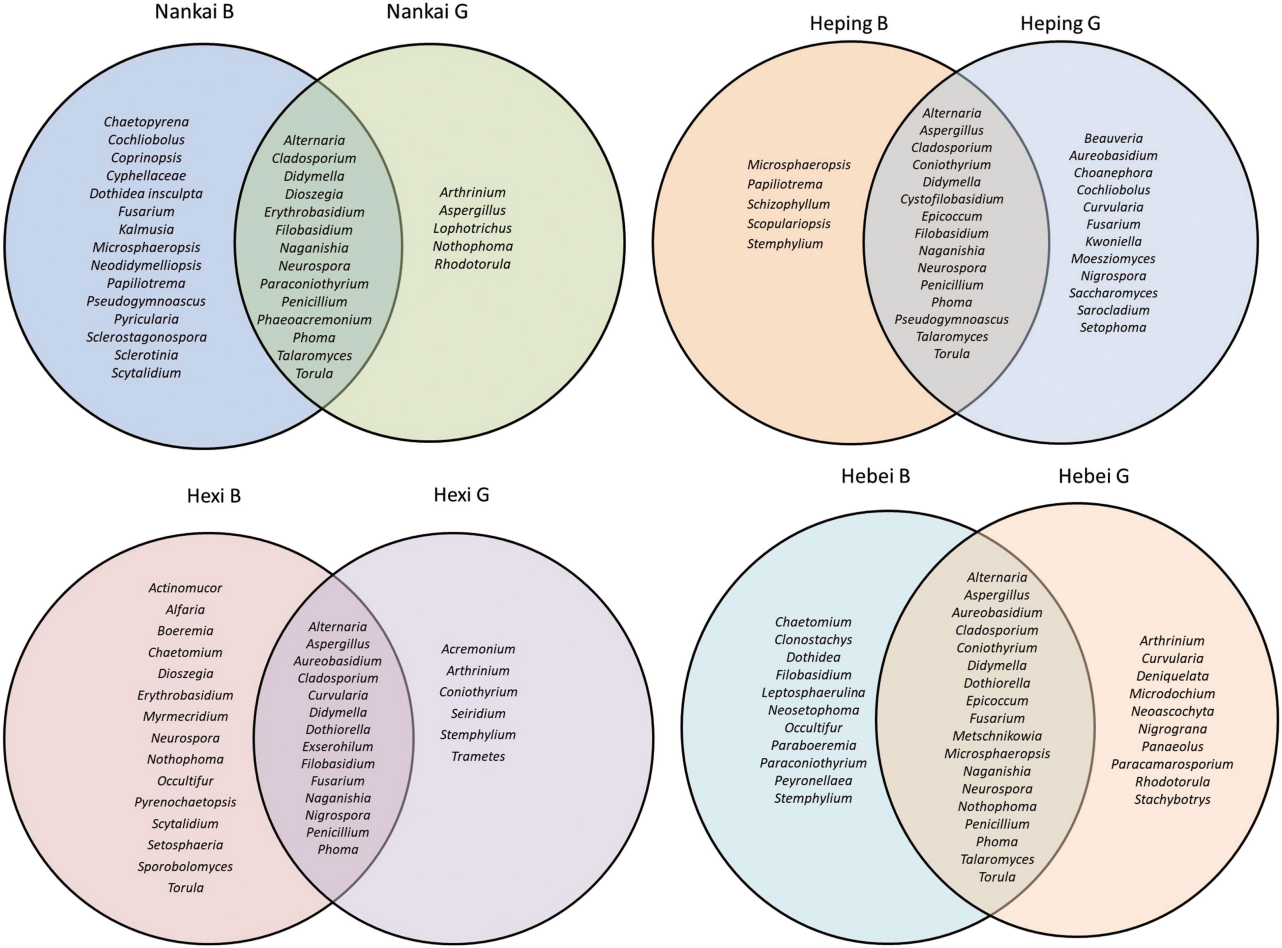
Venn Diagrams showing the diversity of fungal genera isolated at each district and site (green vs. busy).

Different fungal genera were observed at the investigated city districts and sites. The overlapping part in the Venn diagrams presents the fungal genera observed in both green and busy sites (Figure 4). A total of 82 fungal genera were recorded during the one-year sampling. Among them, 31 genera were present in both green and busy sites whereas 21 and 30 fungal genera were only found in green and busy sites, respectively (Supplementary Figure 3).

The total fungal colony concentration recorded during the whole study from April 2020 to March 2021 ranged from 0 CFU/m^3^ to 340 CFU/m^3^ and varied from different locations and months (Table 1, Supplementary Table 1). Looking at the different locations, the highest total fungal colony concentration in Nankai District was 300 CFU/m^3^, recorded in Nankai G site in the month of May, while the lowest value was recorded in Nankai B site, with no culturable fungi isolated in February. Similarly, in Heping District, the green site (Heping G) showed the highest fungal colony concentration (290 CFU/m^3^) in May, while the least total fungal concentration (10 CFU/m^3^) was reported in the busy site (Heping B) in February. In Hexi District, the highest concentration of fungal colonies (220 CFU/m^3^) was recorded in Hexi B site in the months of May and October, while the lowest value was 10 CFU/m^3^, recorded in Hexi B in July. For Hebei District, the highest and the least concentrations were 340 CFU/m^3^ and 10 CFU/m^3^ recorded in Hebei G in the month of April and February, respectively (Table 1).

**Table 1:** Fungal colony concentration recorded in each site.

Sampling location	Colony concentration (CFU/m^3^)											
	April	May	June	July	August	September	October	November	December	January	February	March
NanKai B	170	230	50	60	50	20	50	70	280	50	0	40
NanKai G	160	300	80	170	70	60	130	60	180	50	10	110
HePing B	170	240	50	100	100	100	100	50	270	50	10	70
HePing G	130	290	210	70	40	100	250	50	150	40	50	30
HeXi B	130	220	170	10	50	80	220	50	180	30	20	110
HeXi G	70	160	90	110	80	120	100	100	80	50	30	30
HeBei B	100	120	120	30	120	110	140	150	110	30	30	70
HeBei G	340	320	90	50	100	220	170	110	70	20	10	60

Sampling district name (Nankai, Heping, Hexi, Hebei) and site (G = Green, B = Busy) are indicated.

Looking at the data collected from all the sampling sites, at whole city level, the total fungal concentration was significantly varying in different months at *p* < 0.05 (Table 2). The highest mean fungal concentration was 235 CFU/m^3^ observed in May, followed by the months of April and December, which had no significant difference from each other, showing a total fungal concentration of 165.00 and 158.75 CFU/m^3^, respectively (Table 2). The fourth highest airborne fungal concentration in Tianjin city was recorded in the month of October with 145.00 CFU/m^3^. The months of June, September, November, August, and July showed an intermediate level of total fungal colonies (107.50, 101.25, 80.00, 76.25, and 75.00 CFU/m^3^, respectively), while the least fungal concentration values were recorded in January, February and March (40.00, 20.00, and 65.00 CFU/m^3^, respectively, Table 2).

**Table 2:** Average fungal concentration recorded at different months across every location.

Sampling month	Colony concentration (CFU/m^3^)			
	Mean	SD	Min	Max
April	158.75^ab^	81.141	70	340
May	235.00^a^	69.282	120	320
June	107.50^bcd^	56.758	50	210
July	75.00^bcd^	50.709	10	170
August	76.25^bcd^	28.754	40	120
September	101.25^bcd^	57.678	20	220
October	145.00^abc^	66.117	50	250
November	80.00^bcd^	36.645	50	150
December	165.00^ab^	79.462	70	280
January	40.00^d^	11.952	20	50
February	20.00^d^	16.036	0	50
March	65.00^cd^	32.071	30	110

Values with the same superscript alphabets are not significantly different based on Tukey HSD. Mean = Average fungal Concentration, SD=Standard Deviation.

Shannon index was used to analyze the taxonomic diversity of fungal community at different months for the whole study period (Table 3). The highest value of Shannon index was 3.217 recorded in the month of December and the lowest was 1.676 observed in August (Table 3).

**Table 3:** Monthly values of Shannon index and evenness.

Sampling month	Index	Evenness
January	2.94	0.966
February	2.54	0.99
March	2.836	0.905
April	2.91	0.818
May	1.87	0.589
June	2.694	0.758
July	2.201	0.735
August	1.676	0.654
September	2.707	0.796
October	3.116	0.839
November	3.123	0.901
December	3.217	0.861

Overall, 47 and 53% of colonies were isolated from busy and green sites, respectively, thus surprisingly showing no significant difference. However, looking at the detailed percentage of fungal strains recorded in each month, July, September, and February showed a significantly higher concentration of airborne fungi in green sites than in busy sites, while in December the results were reversed, with busy site fungal communities being richer than those in green areas (Supplementary Figure 4).

### 3.3. Environmental factors and seasonal variation in airborne fungal communities

**Figure 5:**
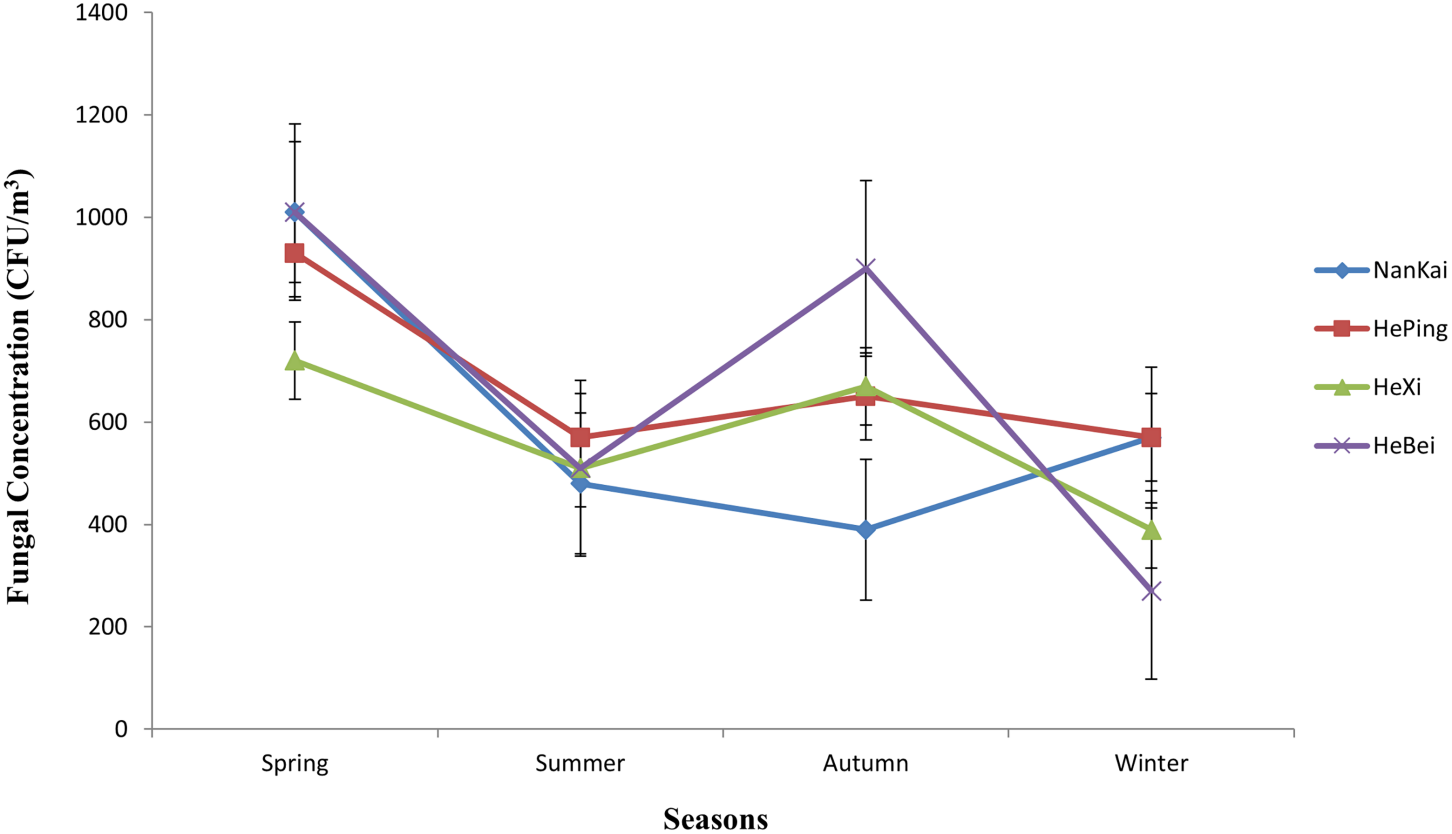
Seasonal variation of airborne fungal concentrations at district level, in Tianjin.

**Figure 6:**
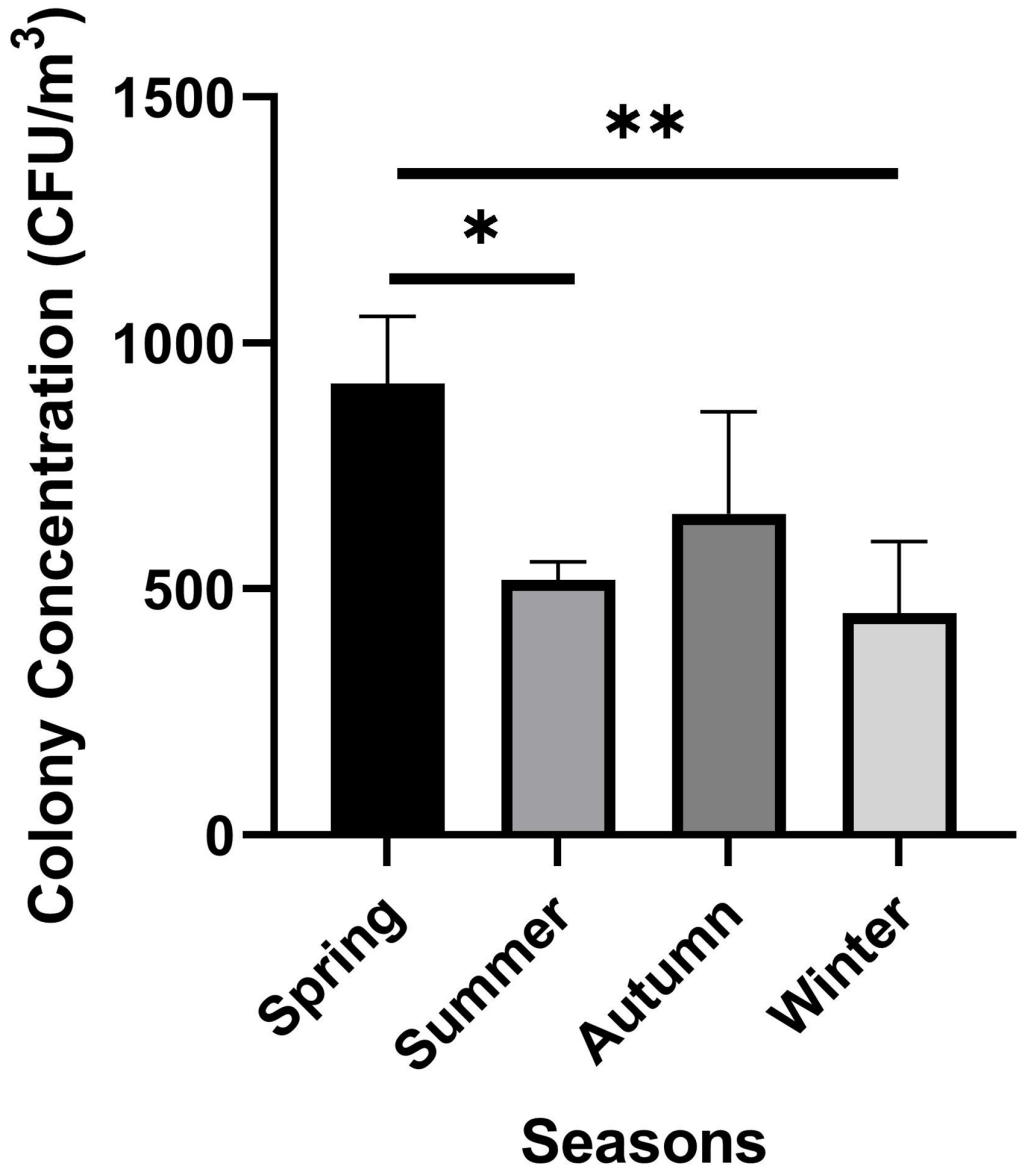
Average colony concentration in different seasons, at city level (*p < 0.05 spring vs. summer, **p < 0.05 spring vs. winter).

A varying fungal concentration was observed for each of the four analyzed Tianjin districts in different seasons (Figure 5). The highest colony concentration was recorded in spring (from March to May) as 3,670 CFU/m^3^, followed by 2,610 and 2070 CFU/m^3^ found in autumn (September–November) and summer (June–August), respectively, while the least fungal concentration (1800 CFU/m^3^) was observed in winter season (December–February). At city level, the average fungal concentration varied significantly among seasons (Figure 6), with the biggest difference observed between spring and winter (**p < 0.05). A strong variation in airborne fungal presence was also observed between spring and summer (*p < 0.05), while there was not statistically significant variation between other seasons (Figure 6).

Environmental parameters (Temperature, Relative Humidity, Wind speed, and Air Pressure) were correlated with the total fungal colonies observed in all the analyzed locations during each month, based on Pearson Correlation analysis. The temperature showed a high positive correlation with the total fungal concentration observed in September (*r* = 0.853**, *p* < 0.01), while it was negatively correlated with the total fungal concentration in the month of November (*r* = −0.7718*, *p* < 0.05). The relative humidity showed a high positive correlation with the total fungal concentration in the month of January (*r* = 0.720*, *p* < 0.05), and a high negative correlation in September (*r* = −0.819*, *p* < 0.05, Table 4). For wind speed, a high positive correlation with fungal concentration was observed in August (*r* = 0.724*, *p* < 0.05), whereas a high negative correlation was observed in the months of September (*r* = −0.774*, *p* < 0.05) and November (*r* = −0.817*, *p* < 0.05). The air pressure showed negative (*r* = −0.819*, *p* < 0.05) and positive correlation (*r* = 0.740*, *p* < 0.05) with fungal concentration in the month of November and December, respectively (Table 4). At season level, temperature showed a high negative correlation with total fungal colony concentration in winter, a medium positive correlation in the autumn season, and a low negative correlation in summer and spring (Table 5). Relative humidity showed a high negative correlation with fungal concentration in autumn season (*r* = −0.763*, *p* < 0.05), while medium positive correlation in winter, and low positive correlation in spring and summer seasons were observed. Wind speed and air pressure both showed a strong positive correlation with fungal concentration in winter and a strong negative correlation in autumn (Table 5).

**Table 4:** Pearson correlation analysis between fungal concentration and environmental factors during 1 year sampling.

	April	May	June	July	August	September	October	November	December	January	February	March
Temp	−0.422	0.101	−0.16	0.269	−0.069	0.853**	0.599	−0.771*	−0.462	−0.697	0.622	0.367
RH	0.365	0.355	−0.19	−0.361	−0.304	−0.819*	−0.208	0.614	0.169	0.720*	−0.41	−0.259
Wind	0.442	−0.134	0.027	−0.527	0.724*	−0.774*	0.353	−0.817*	0.696	−0.527	0.093	−0.184
Air Pressure	−0.317	0.349	0.027	0.527	−0.603	−0.706	−0.294	−0.819*	0.740*	0.689	−0.128	−0.289

* Correlation significant at 0.05 level (2-tailed).

** Correlation significant at 0.01 level (2-tailed).

**Table 5:** Pearson correlation analysis between fungal concentration and environmental factors during the four analyzed seasons.

	Spring	Summer	Autumn	Winter
Temp	−0.197	−0.241	0.382	−0.532
RH	0.223	0.215	−0.763*	0.277
Wind	0.325	−0.069	−0.827*	0.815*
Air Pressure	−0.018	0.010	−0.800*	0.818*

* Correlation significant at 0.05 level (2-tailed).

** Correlation significant at 0.01 level (2-tailed).

**Figure 7:**
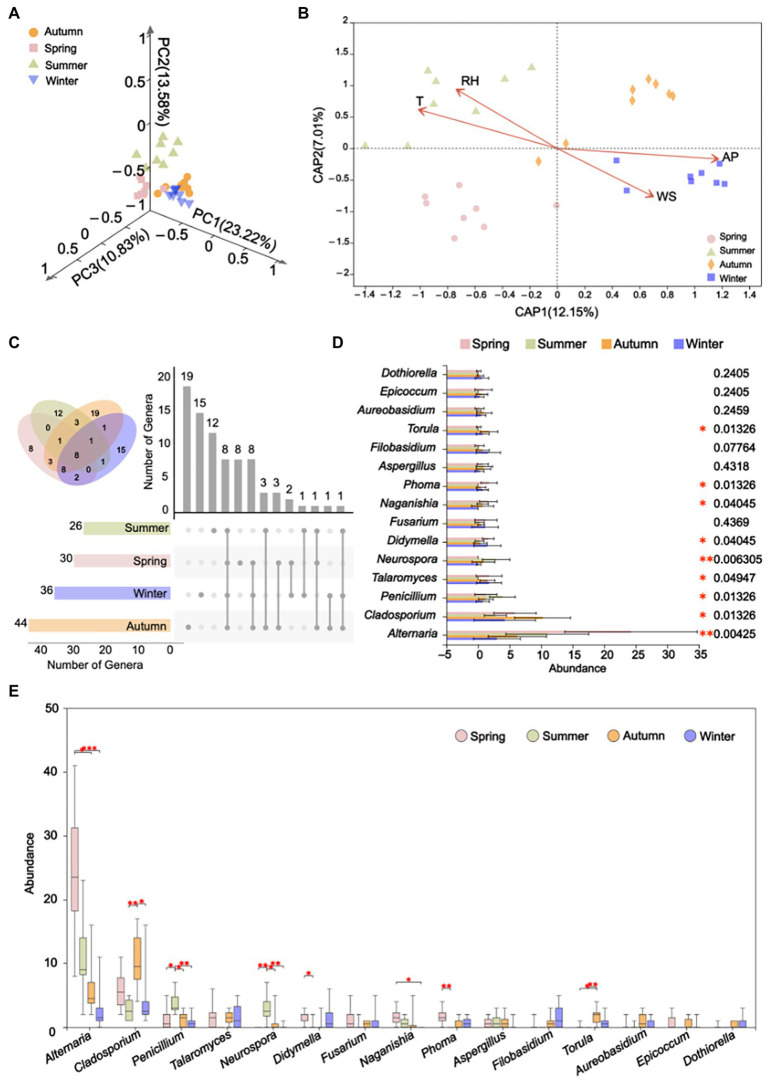
Variances of fungal communities in four sampling seasons and effects of environmental factors on the airborne fungal diversity. **(A)** 3D PCoA plot of airborne fungal communities in four seasons based on Bray–Curtis distance (ANOSIM, *p* = 0.001, *r* = 0.5360). **(B)** Distance-based Redundancy Analysis of the fungal communities, with symbols coded by seasons. **(C)** Number of shared and unique fungal genera among four seasons. The numbers are indicated in the respective circles and bars. **(D)** Comparison of top-fifteen abundant fungal genera in four seasons. Differences in the genera abundance as evaluated by Kruskal-Wallis test. **(E)** Differences of top-fifteen abundant fungal genera between two seasons based on *Post hoc* Dunn’s test. In the sub-figures, T, RH, WS and AP refer to “temperature,” “relative humidity,” “wind speed” and “air pressure,” respectively. The value *p* is indicated as: *p < 0.05, **p < 0.01, ***p < 0.001.

Principal Co-ordinates Analysis demonstrated that airborne fungal communities of Tianjin outdoor environments in spring, summer, autumn and winter seasons were clearly distinguished based on Bray–Curtis distance (ANOSIM *p* = 0.001; Figure 7A). The dbRDA revealed that all four recorded environmental factors, namely temperature, relative humidity, wind speed and air pressure exhibited significantly high correlations (*p* = 0.001) with fungal community composition (Figure 7B). The main influencing factors on fungal community varied in different seasons. For spring and summer, temperature and relative humidity showed relatively higher positive influence on the fungal communities compare with the other two factors, as revealed by the spots representing fungal communities of spring and summer concentrated near the temperature and relative humidity arrows (Figure 7B). By contrast, wind speed and air pressure showed greater positive impacts on the fungal communities in autumn and winter (Figure 7B). Among all the 82 fungal genera detected in the outdoor air of Tianjin, only eight genera were commonly shared by the four different seasons (Figure 7C), including *Alternaria*, *Aspergillus*, *Aureobasidium*, *Cladosporium*, *Coniothyrium*, *Didymella*, *Fusarium*, and *Penicillium*. More diverse fungal genera were found in autumn with 44 fungal genera detected, followed by winter (36 genera), spring (30), and summer (26). Autumn also held the highest number of unique fungal genera (19) in four seasons (Figure 7C). According to the Kruskal-Wallis test, several abundant fungal genera were overall significantly differentiated from four seasons (Figure 7D), including *Alternaria*, *Neurospora* (*p* < 0.01), *Cladosporium*, *Penicillium*, *Talaromyces*, *Didymella*, *Naganishia*, *Phoma*, and *Torula* (*p* < 0.05). Looking at more detailed differences in terms of fungal abundance between each pair of seasons based on *Post hoc* Dunn’s test, eight fungal genera were found to be significantly different between at least one pair of seasons. For instance, *Alternaria*, which was the most dominant genus in the outdoor air of Tianjin, was much more abundant in spring than in winter (*p* = 0.0002) and autumn (*p* = 0.0188; Figure 7E).

**Figure 8:**
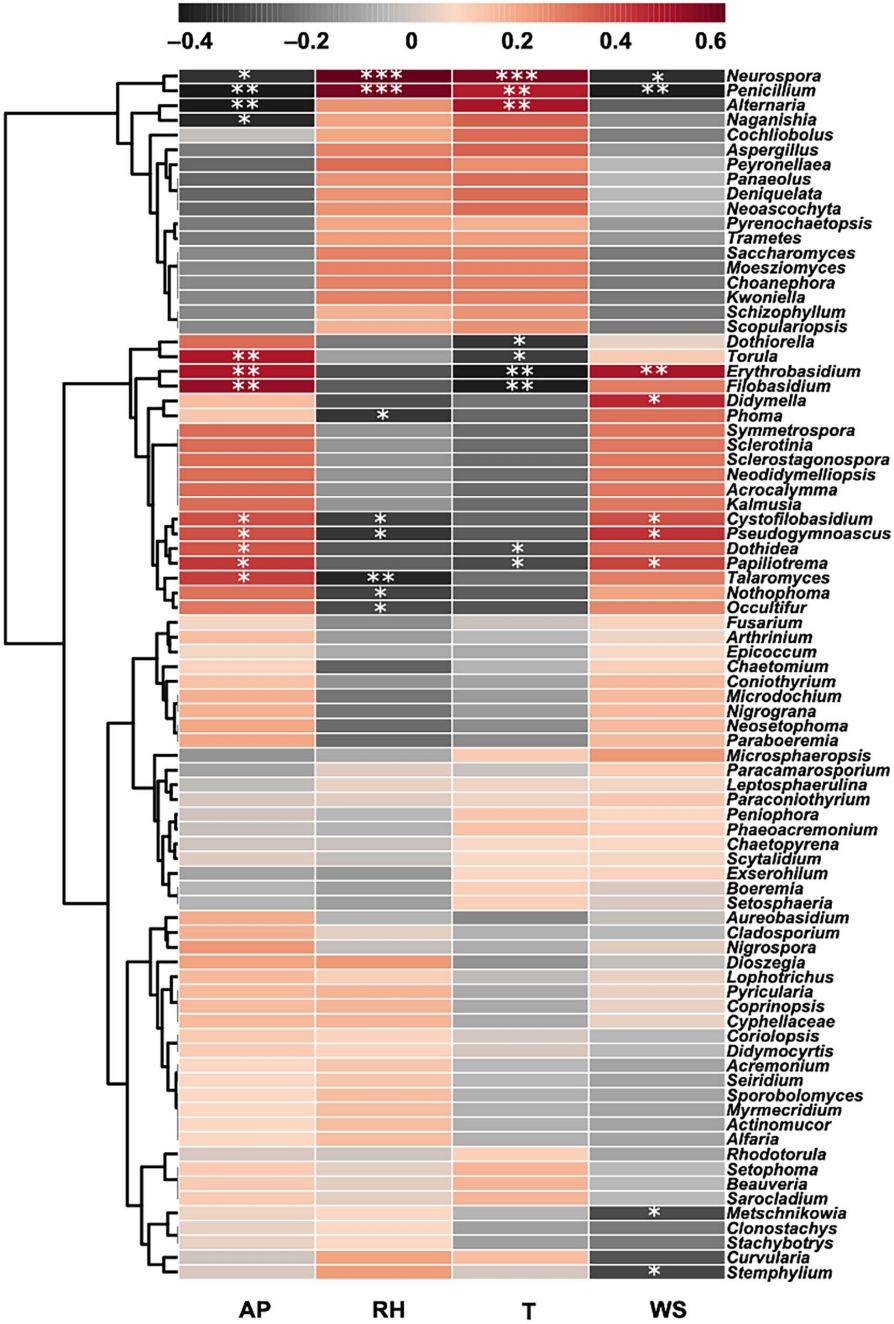
Correlation Heat Map of the detected 82 fungal genera and four environmental factors (AP = Air Pressure, RH = Relative Humidity, T = Temperature and WS = Wind Speed). Different colors infer to Spearman’s correlation coefficients (*r*). The value *p* is indicated as: *p < 0.05, **p < 0.01, ***p < 0.001.

The Correlation Heat Map showed the influences of environmental variables on all 82 detected fungal genera (Figure 8). Among the top 15 abundant genera (i.e., *Alternaria*, *Cladosporium*, *Penicillium*, *Talaromyces*, *Neurospora*, *Didymella*, *Filobasidium*, *Phoma*, *Aspergillus*, *Torula*, *Fusarium*, *Naganishia*, *Aureobasidium*, *Epicoccum*, and *Dothiorella*), 10 genera were significantly positively/negatively correlated with at least one recorded factor, whereas *Cladosporium*, *Aspergillus*, *Fusarium*, *Aureobasidium* and *Epicoccum* did not show notable correlation with any factor. In particular, extremely prominent correlations (*p* < 0.001) were found between *Penicillium* and relative humidity (*r* = 0.5667), and *Neurospora* and both relative humidity (*r* = 0.6283) and temperature (*r* = 0.5582). Besides the abundant fungal genera, other nine genera were also significantly associated with different factors, including *Erythrobasidium*, *Cystofilobasidium*, *Pseudogymnoascus*, *Dothidea*, *Papiliotrema*, *Nothophoma*, *Occultifur*, *Metschnikowia*, and *Stemphylium* (Figure 8, Supplementary Table 2).

**Figure 9:**
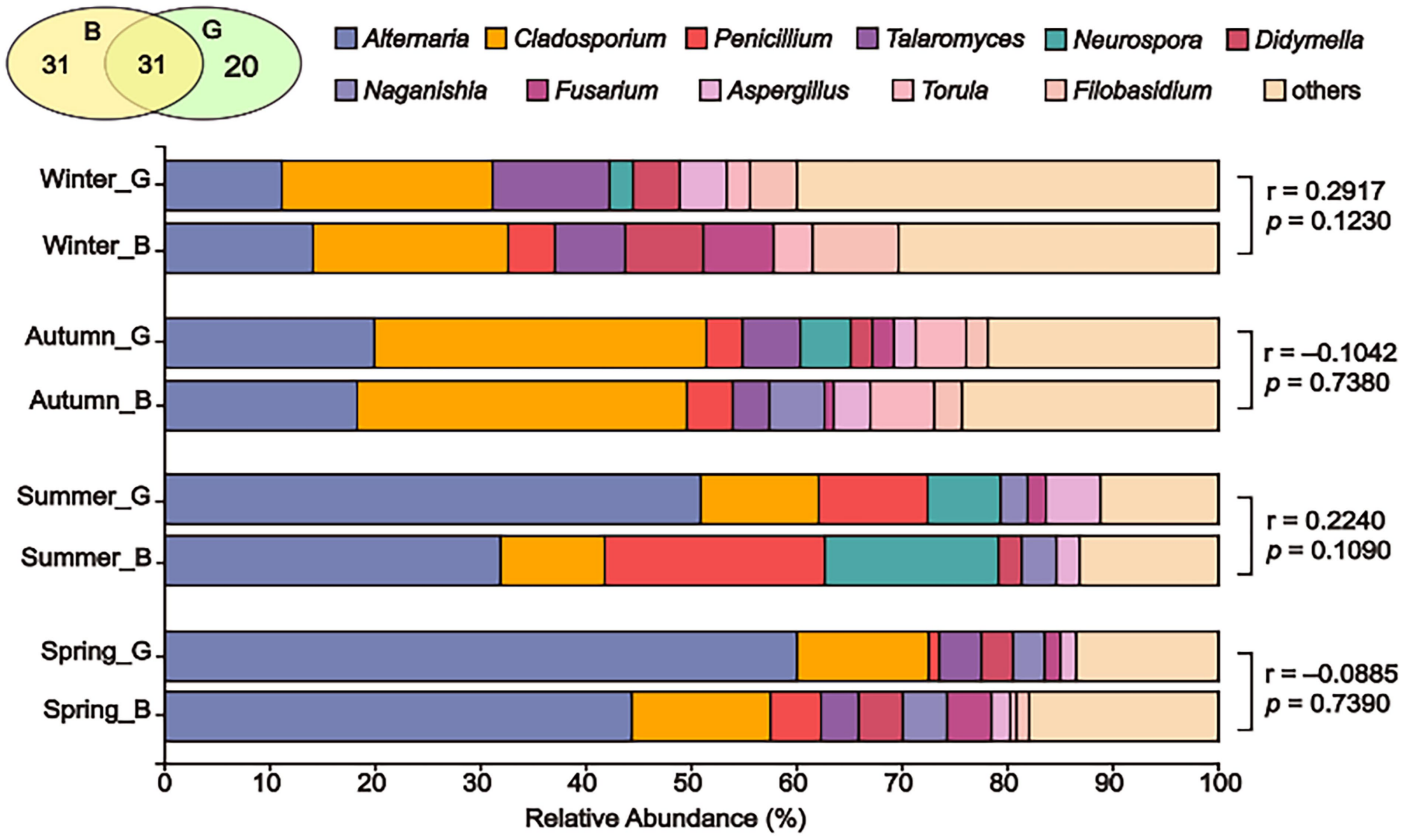
Fungal community composition in the air of green (G) and busy (B) sites from four seasons. The relative abundance of fungal genera <5% were merged as “others” in the bar plots. For each season, statistical differences between fungal communities in green and busy sites were indicated as *p*-values, which were derived from ANOSIM. The number of common and unique airborne fungal genera in green and busy sites of Tianjin was displayed in the Venn diagram.

For each season, no obvious contrasts were found between fungal communities from green and busy sites, as revealed by the ANOSIM *p*-values higher than 0.05 shown in Figure 9. Likewise, no certain fungal genus abundance was significantly different from the two sampling site types on the basis of Wilcoxon rank-sum test (Supplementary Table 3). Overall, 31 fungal genera overlapped in busy and green sites of Tianjin, accounting for 37.80% of total fungal genera (Figure 9), with insignificant differences detected overall in the fungal communities found in busy and green areas (ANOSIM *p* = 0.5410).

## 4. Discussion

The present study, based on large body of data collected during a one-year long sampling, provided valuable information to understand the effect of seasons on the airborne fungal communities characterizing different outdoor environments in one of the largest cities of China, and to disentangle the role of various environmental parameters in shaping the diversity and dynamics of the analyzed fungal communities. The eight sites in four urban districts analyzed in this study showed a high airborne fungal diversity, resulting in the isolation of 1,015 fungal strains belonging to 175 species in 82 genera. Previous studies conducted in other cities of China showed less diverse airborne fungal communities as compared to Tianjin. For example, in a study on outdoor environments performed in 3 different urban areas in Beijing, the number of isolated species was 40, belonging to 14 genera (Fang et al., 2005), while 85 airborne fungal species in 21 genera were isolated during a research conducted in 4 different sampling sites in Hangzhou city (Fang et al., 2019). The high diversity of fungi in the outdoor environments of Tianjin is also confirmed by comparison with the results provided by studies carried out in other countries. For instance, Menezes et al. (2004) isolated a total of 24 fungal genera during a period of 1 year, in a study on the airborne fungi in Fortaleza, Brazil, while Ponce et al. (2013) detected 19 genera of airborne fungi in a study conducted in the city of Mérida, Mexico, over the three most characteristic seasons in the studied region, the cold fronts, the dry, and the rainy seasons. The fungal concentration in the analyzed outdoor environments varied significantly in the different sampling sites over the span of a year. Overall, fungal concentrations ranging from 0 to 340 CFU m^−3^ were recorded across the eight distinct sites investigated in Tianjin. The broad variation in fungal concentrations might be linked to micro-environmental and meteorological factors, as well as different climatic conditions throughout the year (Adhikari et al., 2004; Anees-Hill et al., 2022). Similar variations in fungal concentrations of airborne fungi have been recorded in many studies across different cities in various countries (Di Giorgio et al., 1996; Rosas et al., 1997; Shelton et al., 2002; Fang et al., 2019; Kalyoncu, 2019). For example, Ekmekci (2008) in a study on outdoor fungal concentration in Manisa City, Turkey, recorded values ranging between 151 and 569 CFU m^−3^. Shelton et al. (2002), in a research conducted in different outdoor environments of U.S., found that the fungal concentration varied dramatically from levels below the detection limit to >10,000 CFU m^−3^. Fernández-Rodríguez et al. (2014) reported values of outdoor airborne fungal concentration from 5 to 2,590 CFU m^−3^ within Badajoz City, in Spain. In China, a fungal concentration ranging between 24 and 13,960 CFU/m^3^ was recorded by Fang et al. (2005), in a study carried out in outdoor environments of Beijing, whereas a fungal concentration from <12 to 8,767 CFU m^−3^ was observed in air environments of Hangzhou (Fang et al., 2019). Peak concentrations of airborne fungal spores were observed in Bratislava, Slovakia, during summer and early autumn, whereas minimum concentrations were detected in winter and spring (Ščevková and Kováč, 2019). Monitoring of anamorphic fungal spores in the atmosphere of Madeira, Portugal, during 2003–2008, showed the highest concentrations of fungi in the periods of April–June and September–November, while the lowest levels of spore presence were recorded in December–February (Sousa et al., 2015). These varying levels of fungal concentration recorded in the outdoor environments of different cities across the world were attributed to the influence of different environmental parameters, including human activities, vegetation presence, traffic flow and seasonal variations (Pasanen et al., 1996; Awad, 2002; Adhikari et al., 2004).

In the present work, the most abundant fungal genera detected in the analyzed sampling environments were *Alternaria, Cladosporium* and *Penicillium.* These fungal genera have been similarly identified as dominant taxa in various air environments analyzed in previous studies (Fang et al., 2005; Aira et al., 2007; Ekmekci, 2008; Martinez-Bracero et al., 2022). For instance, *Cladosporium*, *Penicillium*, *Aspergillus*, and *Alternaria* were identified as the most abundant fungal genera in the outdoor environments of Manisa, Turkey (Ekmekci, 2008). In Beijing, China, the genera *Cladosporium*, *Alternaria*, *Penicillium* and *Aspergillus* dominated the airborne fungal communities analyzed by Fang et al. (2005). *Cladosporium* was recorded as the most dominant fungal genus in the outdoor air of Badajoz, Spain, followed by *Penicillium*, *Aspergillus* and *Alternaria* (Aira et al., 2007). The genera *Cladosporium*, *Leptosphaeria*, and *Alternaria* were dominant in the airborne fungal communities of urban outdoor environments in Poland (Kasprzyk et al., 2021). In the present study, *Alternaria*, comprising 35% of the total isolated strains, was the dominant fungal genus recorded during the whole year of sampling. Different concentrations of *Alternaria* fungi were linked to increased respiratory symptoms and airway responsiveness by Downs et al. (2001), who described the analyzed fungal genus as a major cause of asthma. In the latter study, the most frequently found *Alternaria* species was *A. alternata* (Downs et al., 2001), which was also the most common taxon in the present entire research. This fungal species has been primarily associated with the development of immunoglobulin E (IgE)-mediated respiratory illnesses (Gabriel et al., 2016; Quesada, 2022). Clinically, *A. alternata* has been linked to asthma (Bush and Prochnau, 2004; Pulimood et al., 2007; Mohammad and Khalil, 2022), allergic rhinosinusitis (Schell, 2000; Sanchez et al., 2022), hypersensitivity pneumonitis (Ogawa et al., 1997), oculomycosis (Ozbek et al., 2006), onychomycosis (Romano et al., 2001), skin infections (Mayser et al., 2002), and Allergic Bronchopulmonary Mycosis (ABPM; Singh and Denning, 2012; Chowdhary et al., 2012).

*Cladosporium* was found to be the second most frequent genus in the analyzed Tianjin outdoor environments, while *C. cladosporioides* was the second most frequently occurring species. *Cladosporium* species are generally saprophytic, but they are also occasionally linked to opportunistic infections in humans and animals (Sandoval-Denis et al., 2015; Martins, 2022). The genus *Cladosporium* was identified as the most abundant fungal taxon in nasal microbiota sampled from 135 people, either healthy or allergic, in a study conducted in Barcelona, Spain, where *C. herbarum* and *C. cladosporioides* were the dominant species (23.6%; Sellart-Altisent et al., 2007). *Cladosporium cladosporioides* was identified as a cause of subcutaneous phaeohyphomycosis in some clinical studies (Gugnani et al., 2000, 2006; Sang et al., 2012). In a study performed by Ma et al. (2013) in China, *C. cladosporioides* was identified as a cause of phaeohyphomycotic dermatitis in giant pandas. The genus *Penicillium* showed the third greatest concentration in the present study. *Penicillium* species are among the most common allergenic fungi (Pashley and Wardlaw, 2021; Hughes et al., 2022). However, the true impact of these ubiquitous fungal species on atopic respiratory illnesses and the rate of allergic sensitization to *Penicillium* antigens are still largely unknown (Chou et al., 2003). Although the WHO–IUIS Allergen Nomenclature Sub-committee has recognized multiple allergens from various *Penicillium* species, no major allergen components specific to this genus have been recorded. In a study conducted on infants with asthma, Gent et al. (2002) reported that the higher *Penicillium* concentrations were linked to a higher likelihood of wheezing and chronic cough.

Seasonal changes in the environment are known to determine important variations in outdoor airborne fungal communities (Larsen, 1981; Kumar et al., 2021; Núñez et al., 2021; Yuan et al., 2022). We found a significant difference in fungal concentration in the analyzed environments in Tianjin, with the highest level recorded in spring and the lowest in winter. This seasonal variation in the airborne fungal community could be linked with vegetation dynamics (Picco and Rodolfi, 2000; Kasprzyk et al., 2021). In a study carried out by Picco and Rodolfi (2000), the majority of fungal spores in the air were thought to originate from vegetation rather than soil, and more specifically from phylloplane. The high fungal concentration that we observed in spring could be due to the vigorous vegetation growth characterizing the majority of plant species present in Tianjin territory in this season, which develop new leaves after the winter dormancy imposed by the local climate. Similarly, we may hypothesize that the relatively high fungal concentration recorded in autumn, the second highest in the whole study, was enhanced by vegetation sources and by the seasonal degradation of plant debris, which represent suitable substrate for the growth of numerous saprotrophic fungi, as previously observed by Larsen (1981). Similarly, Tignat-Perrier et al. (2020) observed that fungal saprotrophs increased their relative abundance within the airborne microbial communities of Puy de Dôme (France) during the autumn season, when dead and decomposing organic matter covered terrestrial surfaces. Besides, in autumn and spring, air temperature and moisture in the environment may be more favorable for airborne fungal germination, growth, and proliferation than in winter, when the extremely dry weather, combined with low air temperatures is likely to reduce the fungal growth (Fang et al., 2015; Patel et al., 2018; Núñez et al., 2021).

The present accurate and long-term analysis of Tianjin air environments clarified the relative influence on the studied airborne fungal communities of meteorological factors, including temperature, relative humidity, wind speed, and air pressure, which are known to have a significant impact on the diversity and distribution of fungi in urban environments (Larsen, 1981). We observed that air temperature and moisture had a greater effect on the studied fungal communities during the spring and summer seasons, whereas wind speed and air pressure had a stronger influence in autumn and winter. These findings are in agreement with the results reported by Fang et al. (2005) from outdoor environments in Beijing, China. In the latter study the air temperature and humidity in summer and autumn were found to be more suitable for fungal growth and supported a better propagation of airborne fungi as compared to the winter season Fang et al. (2005). Similarly, the air temperature was found to enhance fungal growth and germination in Hangzhou, in all seasons except in winter (Fang et al., 2019). Clear seasonal variations of the influence of various environmental factors on airborne fungal communities was also observed in a 2 years survey on the composition, variability, and sources of microbes in the air of Madrid, Spain (Núñez et al., 2021). Concerning the relatively stronger influence that wind and atmospheric pressure showed in the present study on the analyzed fungal communities during autumn and winter, it is interesting to notice that this influence produced contrasting effects. On the one hand, the diversity of fungi recorded in autumn and winter, yielding 44 and 36 genera, respectively, was higher than in the other seasons. This finding is in agreement with previous reports on the aiding effect of wind in the release, suspension, and dissemination of fungal spores from fruit bodies and conidiophores produced by fungi growing on different substrates, such as soil and plant surfaces, into the air (Picco and Rodolfi, 2000; Giri et al., 2008). Besides, Pyrri and Kapsanaki-Gotsi (2017) observed that elevated atmospheric pressure may promote the suspension of fungal spores in the air, by preventing their sedimentation. On the other hand, the total concentration of airborne fungi was much lower in winter than in summer, and it was the lowest of the whole year of study in winter. Similarly, the highest diversity of fungi detected in Tianjin air in autumn did not support high level of fungal community concentration, which was much lower in this season compare with spring. We can understand these contrasting effects of the environmental conditions on the increased diversity and decreased concentration of airborne fungi, by looking at the results of the present work more in detail. It is noteworthy to observe that in autumn, which showed the highest fungal diversity, 19 out the 44 collected fungal genera were unique to this season, while the second highest number of genera exclusively found in a single season was recorded in winter (15 out of 36). It is possible that the higher diversity of culturable fungi revealed by the results of this study in winter and autumn was sustained by the presence of fungal taxa that were able to tolerate the environmental conditions recorder in these seasons, even if they could not achieve a high distribution in the environment and therefore a high abundance in the air. Moreover, we cannot exclude the possibility that fungal species found as dominant in spring and summer reduced the chance to detect less abundant fungi, which were overgrown on the culture medium. This could be, for instance, the case of *Alternaria*, which was the most dominant fungal genus in the analyzed outdoor air of Tianjin, while it was much more abundant in spring than in winter and autumn. The remarkably dominant presence in the air of *Alternaria* viable particles during spring could be explained by the simultaneous large availability of newly emerged plant organs, particularly leaves, in this season, given that *Alternaria* fungi are recognized as common plant pathogens (Rotem, 1994). In a previous study performed by our research group on the indoor airborne fungal communities in a research and teaching building of Tianjin University (Lu et al., 2022), it was hypothesized that the high concentration of *Alternaria* fungi recorded both inside and outside the investigated building could be sustained by long distance transport of *Alternaria* conidia originating from crops and orchards situated in the surrounding territory of Tianjin. Similarly, the atmospheric concentration of *Alternaria* spores in Badajoz, South-west Spain, was correlated to different types of agricultural land in the surrounding areas (Fernández-Rodríguez et al., 2014), while airborne microbial communities in a mid-altitude mountain area of France showed increased relative abundance of fungal phytopathogens and leaf-associated fungi during the spring and summer periods when the crop plants grew and trees were green (Tignat-Perrier et al., 2020). Skjøth et al. (2012) found that agricultural crop farming in Denmark and Central Europe was associated with the presence of spores from allergenic fungal species in Copenhagen, particularly during harvest.

According to Awad (2002), one of the most significant sources of fungal air contamination is vegetation, while several studies showed a significant influence of air pollution on fungal diversity and concentration, although with controversial results (Dong et al., 2016; Yan et al., 2016; Zhou et al., 2021; Lu et al., 2022). In this study, we attempted to better understand the influence of vegetation presence and air pollution on airborne fungi in urban environments, by comparing the structure of the fungal communities present in green vs. busy areas, in different Tianjin districts. Surprisingly, statistical analysis showed no significant difference in the concentration of airborne fungi in the two analyzed environments. Numerous studies have previously revealed a strong link between vegetation coverage and airborne fungal diversity and concentrations in outdoor environments (Picco and Rodolfi, 2000; Fang et al., 2005, 2015, 2019). For example, in a research performed in Beijing, Fang et al. (2005) found that airborne fungi in areas characterized by high vegetation cover were more diverse than those in densely inhabited and polluted locations. A study conducted in Rzeszow, south-eastern Poland, showed that the vegetation in the three analyzed urban parks was a source of a huge number of spores from various strong allergenic fungal genera, including *Alternaria* and *Cladosporium* (Kasprzyk et al., 2021). Previous studies have also found that green areas with a wide variety of plants had significantly greater fungal concentrations as compared to areas with lower plant diversity (Picco and Rodolfi, 2000; Fang et al., 2005, 2015, 2019). Anees-Hill et al. (2022) described a strong link between airborne fungal diversity and composition and quantity of local vegetation, which provides a resource for fungal growth, sporulation and spore release. The plant leaf surface (phylloplane) is a habitat characterized by intricate interactions between the host plant and the phyllosphere microorganisms that can access nutrients secreted from the leaf (Inacio et al., 2002). This can allow plant saprophytic and parasitic fungi to proliferate (Picco and Rodolfi, 2000; Kasprzyk et al., 2021) and significantly contribute to the concentration of air fungal spores (Levetin and Dorsey, 2006). The discrepancies between our findings and the above-mentioned previous studies could be explained by the relatively small dimensions of the green areas in Tianjin. This could potentially result in a significant influence from the nearby busy areas on the airborne fungal communities present in the city parks, thus attenuating the effect of environmental factors, such as the vegetation presence, which were expected to characterize the investigated green areas, and consequently produce a strong influence on their airborne fungal diversity and concentration.

## 5. Conclusion

The present study, based on data collected over the course of a whole year, allowed us to better comprehend the seasonal variations of the outdoor fungal communities in Tianjin and to link the diversity and concentration of recorded fungi to various environmental factors. The composition and structure of the investigated airborne fungal communities appeared clearly different in the four studied seasons due to the substantial impact of variations in temperature, relative humidity, wind speed, and air pressure. In particular, temperature and relative humidity exerted a stronger positive effect on the diversity and concentration of airborne fungi in spring and summer, while wind speed and air pressure showed a higher positive effect in autumn and winter, which were characterized by a significantly more diverse fungal community, constituted by a higher number of taxa, although with lower total concentration compare to the other seasons. The comparison between airborne fungal communities from green and busy sites did not show significant differences over the four analyzed seasons, not even during the period of intense plant growth, when we expected a strong impact of the vegetation presence on the analyzed fungi. It is possible that the aforementioned effect of vegetation was attenuated by the influence of the surrounding busy environments, due to the relatively small size of green areas in Tianjin. In this respect, the analysis of airborne fungi could be used as a proxy for the general characterization of urban environments and, in the specific case of the present study, it may suggest the necessity to include large parks in the city development plan, in order to provide the opportunity for the citizens to find areas with better air quality within the densely populated districts of one of the largest cities of China. The prevalence of fungal genera such as *Alternaria*, *Cladosporium*, and *Penicillium*, which are proven to have potentially harmful health impacts on humans, needs further attention and monitoring in order to assess the risk of diseases associated with these airborne fungi. The results of this study provide valuable information on the factors shaping outdoor fungal diversity and community structure in urban areas, and may serve as a reference for air quality management, microbial contamination control, and prevention of airborne fungi related diseases.

## Data availability statement

The original contributions presented in the study are included in the article/Supplementary materials, further inquiries can be directed to the corresponding author.

## Author contributions

LP conceived the study. Samples were collected by YN and XW. The experiments were designed and supervised by LP. Laboratory experiments and analysis were performed by YN. Results were analyzed by YN, XW, and LP. YN prepared the original draft under the guidance and critical review of LP. While XW also contributed to write and review the manuscript. All authors contributed to the article and approved the submitted version.

## Conflict of interest

The authors declare that the research was conducted in the absence of any commercial or financial relationships that could be construed as a potential conflict of interest.

## Publisher’s note

All claims expressed in this article are solely those of the authors and do not necessarily represent those of their affiliated organizations, or those of the publisher, the editors and the reviewers. Any product that may be evaluated in this article, or claim that may be made by its manufacturer, is not guaranteed or endorsed by the publisher.

## Footnotes

^1^ https://www.wunderground.com

^2^ http://www.ncbi.nlm.nih.gov/Blast.cgi

^3^ https://github.com/marbl/Krona/wiki/KronaTools
