# Supplementary material for: Seasonal variation of airborne fungal diversity and community structure in urban outdoor environments in Tianjin, China
Source: Front Microbiol. 2023 Jan 9;13:1043224. doi: 10.3389/fmicb.2022.1043224 (PMC9869124; doi:10.3389/fmicb.2022.1043224)
Supplement: Supplementary file 1 [file Data_Sheet_1.docx]

**Supplementary information**

**Seasonal variation of airborne fungal diversity and community structure in urban outdoor environments in Tianjin, China**

Yumna Nageen^1^, Xiao Wang^1^ and Lorenzo Pecoraro^1^*

^1^School of Pharmaceutical Science and Technology, Tianjin University, Tianjin, China.

*Correspondence: Lorenzo Pecoraro (telephone: +86 18520824550, e-mail: lorenzo.pecoraro@tju.edu.cn) School of Pharmaceutical Science and Technology, Tianjin University, 92 Weijin Road, Nankai District, Tianjin 300072, China.

**Supplementary Table 1**. Fungal Concentration (CFU/m^3^) & Environmental factors recorded in each sampling site at the time of sampling.

| **Year** | **Month** | **Fungal Concentration (CFU/m^3^) & Environmental Factors** | **Sampling sites** | | | | | | | |
| --- | --- | --- | --- | --- | --- | --- | --- | --- | --- | --- |
|  |  |  | **Nankai B** | **Nankai G** | **Heping B** | **Heping G** | **Hexi B** | **Hexi G** | **Hebei B** | **Hebei G** |
| **2020** | **April** | Fungal Conc. (CFU/m^3^) | 170 | 160 | 170 | 130 | 130 | 70 | 100 | 340 |
|  |  | Temperature (°C) | 13.2 | 12.6 | 14.28 | 12.96 | 14.4 | 11.88 | 12.6 | 10.92 |
|  |  | Humidity (%) | 27.6 | 18 | 13.92 | 20.76 | 15.48 | 19.56 | 21.6 | 24.84 |
|  |  | Wind speed (Km/h) | 11 | 11 | 13 | 13 | 9 | 9 | 13 | 13 |
|  |  | Air pressure (MPa) | 29.99 | 29.99 | 29.96 | 29.96 | 29.93 | 29.93 | 29.84 | 29.84 |
|  | **May** | Fungal Conc. (CFU/m^3^) | 230 | 300 | 240 | 290 | 220 | 160 | 120 | 320 |
|  |  | Temperature (°C) | 23.88 | 25.7 | 24 | 24 | 22.2 | 24 | 25.2 | 24.6 |
|  |  | Humidity (%) | 50 | 51.5 | 54 | 53.2 | 44 | 48 | 47 | 46.7 |
|  |  | Wind speed (Km/h) | 9 | 9 | 9 | 9 | 9 | 9 | 13 | 13 |
|  |  | Air pressure (MPa) | 29.73 | 29.73 | 29.73 | 29.73 | 29.7 | 29.7 | 29.67 | 29.67 |
|  | **June** | Fungal Conc. (CFU/m^3^) | 50 | 80 | 50 | 210 | 170 | 90 | 120 | 90 |
|  |  | Temperature (°C) | 26.41 | 17.28 | 13.36 | 19.93 | 14.86 | 18.78 | 20.74 | 23.85 |
|  |  | Humidity (%) | 62 | 63.86 | 66.96 | 65.97 | 54.56 | 59.52 | 58.28 | 57.91 |
|  |  | Wind speed (Km/h) | 7 | 7 | 7 | 7 | 7 | 7 | 6 | 6 |
|  |  | Air pressure (MPa) | 29.67 | 29.67 | 29.67 | 29.67 | 29.67 | 29.67 | 29.64 | 29.64 |
|  | **July** | Fungal Conc. (CFU/m^3^) | 60 | 170 | 100 | 70 | 10 | 110 | 30 | 50 |
|  |  | Temperature (°C) | 30.5 | 32.2 | 32.5 | 30.9 | 32.2 | 31.6 | 30.8 | 32.4 |
|  |  | Humidity (%) | 64.5 | 54.6 | 52.5 | 58.8 | 50 | 56.5 | 84.2 | 62.2 |
|  |  | Wind speed (Km/h) | 7 | 7 | 7 | 7 | 11 | 11 | 11 | 11 |
|  |  | Air pressure (MPa) | 29.73 | 29.73 | 29.73 | 29.73 | 29.7 | 29.7 | 29.7 | 29.7 |
|  | **August** | Fungal Conc. (CFU/m3) | 50 | 70 | 100 | 40 | 50 | 80 | 120 | 100 |
|  |  | Temperature (°C) | 24.6 | 24.1 | 29.5 | 29.8 | 23.4 | 24.2 | 25.9 | 25.3 |
|  |  | Humidity (%) | 41.5 | 51 | 29.1 | 44.5 | 45.8 | 44.8 | 43.2 | 45.8 |
|  |  | Wind speed (Km/h) | 4 | 4 | 4 | 4 | 4 | 4 | 9 | 9 |
|  |  | Air pressure (MPa) | 29.7 | 29.7 | 29.67 | 29.67 | 29.64 | 29.64 | 29.61 | 29.61 |
|  | **September** | Fungal Conc. (CFU/m^3^) | 20 | 60 | 100 | 100 | 80 | 120 | 110 | 220 |
|  |  | Temperature (°C) | 18.9 | 20.4 | 22.5 | 22.8 | 19.1 | 20.5 | 19.9 | 26.8 |
|  |  | Humidity (%) | 60.2 | 51.7 | 40.1 | 30 | 56.8 | 47 | 27.2 | 19.1 |
|  |  | Wind speed (Km/h) | 16 | 16 | 11 | 11 | 9 | 9 | 7 | 7 |
|  |  | Air pressure (MPa) | 30.05 | 30.05 | 30.05 | 30.05 | 30.02 | 30.02 | 29.99 | 29.99 |
|  | **October** | Fungal Conc. (CFU/m^3^) | 50 | 130 | 100 | 250 | 220 | 100 | 140 | 170 |
|  |  | Temperature (°C) | 20.11 | 20.5 | 19.5 | 25.5 | 19.7 | 20.2 | 19.4 | 20.1 |
|  |  | Humidity (%) | 30.51 | 30.2 | 33.8 | 27 | 32 | 33.7 | 40 | 37.9 |
|  |  | Wind speed (Km/h) | 2 | 2 | 4 | 4 | 7 | 7 | 7 | 7 |
|  |  | Air pressure (MPa) | 30.38 | 30.38 | 30.35 | 30.35 | 30.29 | 30.29 | 30.26 | 30.26 |
|  | **November** | Fungal Conc. (CFU/m^3^) | 70 | 60 | 50 | 50 | 50 | 100 | 150 | 110 |
|  |  | Temperature (°C) | 1.6 | 3.5 | 2.9 | 3.9 | 2.5 | 3.2 | 0 | 0 |
|  |  | Humidity (%) | 38 | 37.7 | 37.7 | 38.2 | 37.4 | 41.1 | 39 | 39 |
|  |  | Wind speed (Km/h) | 11 | 11 | 11 | 11 | 7 | 7 | 4 | 4 |
|  |  | Air pressure (MPa) | 30.67 | 30.67 | 30.64 | 30.64 | 30.61 | 30.61 | 30.52 | 30.52 |
|  | **December** | Fungal Conc. (CFU/m^3^) | 280 | 180 | 270 | 150 | 180 | 80 | 110 | 70 |
|  |  | Temperature (°C) | -7.3 | -8.9 | -7.7 | -7.3 | -7.5 | -7.7 | -6.2 | -5.6 |
|  |  | Humidity (%) | 15.1 | 18.5 | 12.9 | 13 | 14.3 | 15.1 | 13.5 | 12.4 |
|  |  | Wind speed (Km/h) | 29 | 29 | 22 | 22 | 20 | 20 | 16 | 16 |
|  |  | Air pressure (MPa) | 30.73 | 30.73 | 30.73 | 30.73 | 30.7 | 30.7 | 30.7 | 30.7 |
| **2021** | **January** | Fungal Conc. (CFU/m^3^) | 50 | 50 | 50 | 40 | 30 | 50 | 30 | 20 |
|  |  | Temperature (°C) | -3 | -2.7 | 0.3 | 1.4 | -0.8 | 0.4 | 0.6 | 4.7 |
|  |  | Humidity (%) | 20.5 | 21.6 | 17.3 | 13.2 | 17.2 | 17.1 | 14.8 | 12.6 |
|  |  | Wind speed (Km/h) | 11 | 11 | 16 | 16 | 13 | 13 | 16 | 16 |
|  |  | Air pressure (MPa) | 30.58 | 30.58 | 30.55 | 30.55 | 30.49 | 30.49 | 30.49 | 30.49 |
|  | **February** | Colony Conc. (CFU/m^3^) | 0 | 10 | 10 | 50 | 20 | 30 | 30 | 10 |
|  |  | Temperature (°C) | 7.7 | 5.8 | 8.7 | 9.6 | 7.6 | 7.7 | 8.4 | 7.1 |
|  |  | Humidity (%) | 22.1 | 28.5 | 18.2 | 18.6 | 22.7 | 22.6 | 17.3 | 21.2 |
|  |  | Wind speed (Km/h) | 7 | 7 | 7 | 7 | 9 | 9 | 4 | 4 |
|  |  | Air pressure (MPa) | 30.29 | 30.29 | 30.29 | 30.29 | 30.23 | 30.23 | 30.2 | 30.2 |
|  | **March** | Colony Conc. (CFU/m^3^) | 40 | 110 | 70 | 30 | 110 | 30 | 70 | 60 |
|  |  | Temperature (°C) | 11 | 10.5 | 11.9 | 10.8 | 12 | 9.9 | 10.5 | 9.1 |
|  |  | Humidity (%) | 12.3 | 15 | 11.6 | 17.3 | 12.9 | 16.3 | 18 | 20.7 |
|  |  | Wind speed (Km/h) | 13 | 13 | 16 | 16 | 9 | 9 | 9 | 9 |
|  |  | Air pressure (MPa) | 30.05 | 30.05 | 30.08 | 30.08 | 30.05 | 30.05 | 30.05 | 30.05 |

Sampling district name (Nankai, Heping, Hexi, Hebei) and site (G = Green, B = Busy) are indicated.

**Supplementary Table 2.** Correlation analysis between fungal abundance and environmental parameters.

| Genera | r | | | |  | *p* | | | |
| --- | --- | --- | --- | --- | --- | --- | --- | --- | --- |
|  | AP | RH | T | WS |  | AP | RH | T | WS |
| *Acremonium* | 0.0585 | 0.1070 | –0.0875 | –0.1369 |  | 0.7506 | 0.5600 | 0.6338 | 0.4551 |
| *Acrocalymma* | 0.3021 | –0.1848 | –0.2821 | 0.2835 |  | 0.0929 | 0.3113 | 0.1178 | 0.1159 |
| *Actinomucor* | 0.0585 | 0.1264 | –0.1070 | –0.1369 |  | 0.7506 | 0.4905 | 0.5600 | 0.4551 |
| *Alfaria* | 0.0585 | 0.1264 | –0.1070 | –0.1369 |  | 0.7506 | 0.4905 | 0.5600 | 0.4551 |
| *Alternaria* | –0.5392 | 0.2300 | 0.4824 | –0.3041 |  | 0.0015 | 0.2054 | 0.0052 | 0.0906 |
| *Arthrinium* | 0.1246 | –0.1631 | –0.0866 | 0.0233 |  | 0.4970 | 0.3724 | 0.6373 | 0.8992 |
| *Aspergillus* | –0.2532 | 0.2616 | 0.3306 | –0.1697 |  | 0.1620 | 0.1482 | 0.0646 | 0.3532 |
| *Aureobasidium* | 0.1745 | –0.0978 | –0.2123 | –0.0648 |  | 0.3394 | 0.5943 | 0.2434 | 0.7247 |
| *Beauveria* | 0.0974 | 0.0097 | 0.1459 | –0.0782 |  | 0.5957 | 0.9579 | 0.4256 | 0.6705 |
| *Boeremia* | –0.0974 | –0.1459 | 0.0875 | –0.0196 |  | 0.5957 | 0.4256 | 0.6338 | 0.9154 |
| *Chaetomium* | 0.0694 | –0.3105 | –0.0904 | 0.0878 |  | 0.7060 | 0.0837 | 0.6227 | 0.6328 |
| *Chaetopyrena* | –0.0390 | –0.0486 | 0.0486 | 0.0587 |  | 0.8323 | 0.7915 | 0.7915 | 0.7498 |
| *Choanephora* | –0.2144 | 0.2626 | 0.2626 | –0.2542 |  | 0.2387 | 0.1465 | 0.1465 | 0.1604 |
| *Cladosporium* | 0.1567 | 0.0011 | –0.1105 | –0.0826 |  | 0.3918 | 0.9952 | 0.5470 | 0.6530 |
| *Clonostachys* | 0.0195 | 0.0486 | –0.1459 | –0.2542 |  | 0.9157 | 0.7915 | 0.4256 | 0.1604 |
| *Cochliobolus* | –0.0560 | 0.1818 | 0.3076 | –0.2389 |  | 0.7607 | 0.3194 | 0.0868 | 0.1879 |
| *Coniothyrium* | 0.1181 | –0.1840 | –0.1399 | 0.1440 |  | 0.5197 | 0.3133 | 0.4449 | 0.4318 |
| *Coprinopsis* | 0.1364 | 0.1459 | –0.1264 | 0.0196 |  | 0.4565 | 0.4256 | 0.4905 | 0.9154 |
| *Coriolopsis* | 0.0974 | 0.0681 | –0.0292 | –0.0782 |  | 0.5957 | 0.7112 | 0.8740 | 0.6705 |
| *Curvularia* | –0.0410 | 0.1944 | 0.1330 | –0.3394 |  | 0.8236 | 0.2862 | 0.4679 | 0.0574 |
| *Cyphellaceae* | 0.1364 | 0.1459 | –0.1264 | 0.0196 |  | 0.4565 | 0.4256 | 0.4905 | 0.9154 |
| *Cystofilobasidium* | 0.3642 | –0.3915 | –0.2936 | 0.3654 |  | 0.0404 | 0.0267 | 0.1029 | 0.0397 |
| *Deniquelata* | –0.2923 | 0.2237 | 0.3015 | –0.0782 |  | 0.1044 | 0.2184 | 0.0935 | 0.6705 |
| *Didymella* | 0.1315 | –0.3465 | –0.2630 | 0.4399 |  | 0.4733 | 0.0520 | 0.1458 | 0.0117 |
| *Didymocyrtis* | 0.0974 | 0.0681 | –0.0292 | –0.0782 |  | 0.5957 | 0.7112 | 0.8740 | 0.6705 |
| *Dioszegia* | 0.1978 | 0.2148 | –0.1800 | –0.0584 |  | 0.2779 | 0.2377 | 0.3243 | 0.7511 |
| *Dothidea* | 0.3527 | –0.3332 | –0.3511 | 0.3053 |  | 0.0477 | 0.0624 | 0.0488 | 0.0893 |
| *Dothiorella* | 0.3048 | –0.2501 | –0.4170 | 0.0141 |  | 0.0898 | 0.1675 | 0.0176 | 0.9390 |
| *Epicoccum* | 0.0427 | –0.1265 | –0.1232 | 0.0474 |  | 0.8166 | 0.4901 | 0.5016 | 0.7967 |
| *Erythrobasidium* | 0.4770 | –0.3425 | –0.5051 | 0.4785 |  | 0.0058 | 0.0550 | 0.0032 | 0.0056 |
| *Exserohilum* | –0.1416 | –0.1718 | 0.0642 | 0.0743 |  | 0.4394 | 0.3471 | 0.7272 | 0.6860 |
| *Filobasidium* | 0.5153 | –0.3186 | –0.4869 | 0.2711 |  | 0.0025 | 0.0755 | 0.0047 | 0.1335 |
| *Fusarium* | 0.0373 | –0.2040 | –0.0491 | 0.0745 |  | 0.8394 | 0.2627 | 0.7898 | 0.6853 |
| *Kalmusia* | 0.3021 | –0.1848 | –0.2821 | 0.2835 |  | 0.0929 | 0.3113 | 0.1178 | 0.1159 |
| *Kwoniella* | –0.2144 | 0.2626 | 0.2626 | –0.2542 |  | 0.2387 | 0.1465 | 0.1465 | 0.1604 |
| *Leptosphaerulina* | –0.0771 | 0.0140 | 0.0280 | 0.0422 |  | 0.6751 | 0.9395 | 0.8792 | 0.8188 |
| *Lophotrichus* | 0.1364 | 0.0875 | –0.0681 | 0.0196 |  | 0.4565 | 0.6338 | 0.7112 | 0.9154 |
| *Metschnikowia* | 0.0280 | 0.0559 | –0.0979 | –0.3654 |  | 0.8790 | 0.7611 | 0.5941 | 0.0397 |
| *Microdochium* | 0.1657 | –0.2626 | –0.1653 | 0.1369 |  | 0.3649 | 0.1465 | 0.3658 | 0.4551 |
| *Microsphaeropsis* | –0.1825 | –0.1289 | 0.0973 | 0.2212 |  | 0.3174 | 0.4821 | 0.5963 | 0.2237 |
| *Moesziomyces* | –0.2144 | 0.2626 | 0.2626 | –0.2542 |  | 0.2387 | 0.1465 | 0.1465 | 0.1604 |
| *Myrmecridium* | 0.0585 | 0.1264 | –0.1070 | –0.1369 |  | 0.7506 | 0.4905 | 0.5600 | 0.4551 |
| *Naganishia* | –0.4480 | 0.1893 | 0.3268 | –0.1967 |  | 0.0101 | 0.2994 | 0.0679 | 0.2807 |
| *Neoascochyta* | –0.2923 | 0.2237 | 0.3015 | –0.0782 |  | 0.1044 | 0.2184 | 0.0935 | 0.6705 |
| *Neodidymelliopsis* | 0.3021 | –0.1848 | –0.2821 | 0.2835 |  | 0.0929 | 0.3113 | 0.1178 | 0.1159 |
| *Neosetophoma* | 0.1852 | –0.2821 | –0.2042 | 0.1369 |  | 0.3103 | 0.1178 | 0.2622 | 0.4551 |
| *Neurospora* | –0.4321 | 0.6283 | 0.5582 | –0.4359 |  | 0.0135 | 0.0001 | 0.0009 | 0.0126 |
| *Nigrograna* | 0.1657 | –0.2626 | –0.1653 | 0.1369 |  | 0.3649 | 0.1465 | 0.3658 | 0.4551 |
| *Nigrospora* | 0.2210 | –0.0639 | –0.1219 | 0.0000 |  | 0.2241 | 0.7284 | 0.5062 | 1.0000 |
| *Nothophoma* | 0.2891 | –0.3852 | –0.3216 | 0.1964 |  | 0.1085 | 0.0295 | 0.0726 | 0.2814 |
| *Occultifur* | 0.2872 | –0.3635 | –0.3356 | 0.2530 |  | 0.1110 | 0.0408 | 0.0604 | 0.1625 |
| *Panaeolus* | –0.2923 | 0.2237 | 0.3015 | –0.0782 |  | 0.1044 | 0.2184 | 0.0935 | 0.6705 |
| *Papiliotrema* | 0.4052 | –0.3088 | –0.3620 | 0.3856 |  | 0.0214 | 0.0855 | 0.0417 | 0.0293 |
| *Paraboeremia* | 0.1852 | –0.2821 | –0.2042 | 0.1369 |  | 0.3103 | 0.1178 | 0.2622 | 0.4551 |
| *Paracamarosporium* | –0.1364 | –0.0097 | –0.0486 | 0.0978 |  | 0.4565 | 0.9579 | 0.7915 | 0.5945 |
| *Paraconiothyrium* | –0.0233 | –0.0058 | 0.0290 | 0.1050 |  | 0.8994 | 0.9748 | 0.8747 | 0.5672 |
| *Penicillium* | –0.5359 | 0.5667 | 0.4583 | –0.4831 |  | 0.0016 | 0.0007 | 0.0083 | 0.0051 |
| *Peniophora* | –0.0390 | –0.0875 | 0.1070 | 0.0587 |  | 0.8323 | 0.6338 | 0.5600 | 0.7498 |
| *Peyronellaea* | –0.2923 | 0.3015 | 0.2432 | –0.0782 |  | 0.1044 | 0.0935 | 0.1799 | 0.6705 |
| *Phaeoacremonium* | –0.0560 | –0.0979 | 0.1119 | 0.0843 |  | 0.7607 | 0.5941 | 0.5422 | 0.6464 |
| *Phoma* | 0.1073 | –0.4179 | –0.2943 | 0.2935 |  | 0.5588 | 0.0173 | 0.1020 | 0.1030 |
| *Pseudogymnoascus* | 0.3644 | –0.4218 | –0.3215 | 0.4146 |  | 0.0403 | 0.0162 | 0.0728 | 0.0183 |
| *Pyrenochaetopsis* | –0.2534 | 0.1848 | 0.1653 | –0.1760 |  | 0.1618 | 0.3113 | 0.3658 | 0.3354 |
| *Pyricularia* | 0.1364 | 0.1459 | –0.1264 | 0.0196 |  | 0.4565 | 0.4256 | 0.4905 | 0.9154 |
| *Rhodotorula* | –0.0140 | –0.0419 | 0.0839 | –0.1405 |  | 0.9393 | 0.8197 | 0.6480 | 0.4430 |
| *Saccharomyces* | –0.2144 | 0.2626 | 0.2626 | –0.2542 |  | 0.2387 | 0.1465 | 0.1465 | 0.1604 |
| *Sarocladium* | 0.0974 | 0.0097 | 0.1459 | –0.0782 |  | 0.5957 | 0.9579 | 0.4256 | 0.6705 |
| *Schizophyllum* | –0.2144 | 0.1653 | 0.2237 | –0.2542 |  | 0.2387 | 0.3658 | 0.2184 | 0.1604 |
| *Sclerostagonospora* | 0.3021 | –0.1848 | –0.2821 | 0.2835 |  | 0.0929 | 0.3113 | 0.1178 | 0.1159 |
| *Sclerotinia* | 0.3021 | –0.1848 | –0.2821 | 0.2835 |  | 0.0929 | 0.3113 | 0.1178 | 0.1159 |
| *Scopulariopsis* | –0.2144 | 0.1653 | 0.2237 | –0.2542 |  | 0.2387 | 0.3658 | 0.2184 | 0.1604 |
| *Scytalidium* | –0.0076 | –0.0620 | 0.0489 | 0.0350 |  | 0.9670 | 0.7361 | 0.7903 | 0.8493 |
| *Seiridium* | 0.0585 | 0.1070 | –0.0875 | –0.1369 |  | 0.7506 | 0.5600 | 0.6338 | 0.4551 |
| *Setophoma* | 0.0974 | 0.0097 | 0.1459 | –0.0782 |  | 0.5957 | 0.9579 | 0.4256 | 0.6705 |
| *Setosphaeria* | –0.0974 | –0.1459 | 0.0875 | –0.0196 |  | 0.5957 | 0.4256 | 0.6338 | 0.9154 |
| *Sporobolomyces* | 0.0585 | 0.1264 | –0.1070 | –0.1369 |  | 0.7506 | 0.4905 | 0.5600 | 0.4551 |
| *Stachybotrys* | 0.0195 | 0.0486 | –0.1459 | –0.2542 |  | 0.9157 | 0.7915 | 0.4256 | 0.1604 |
| *Stemphylium* | –0.0160 | 0.2050 | –0.0255 | –0.3771 |  | 0.9308 | 0.2604 | 0.8896 | 0.0334 |
| *Symmetrospora* | 0.3021 | –0.1848 | –0.2821 | 0.2835 |  | 0.0929 | 0.3113 | 0.1178 | 0.1159 |
| *Talaromyces* | 0.3915 | –0.4592 | –0.2715 | 0.2674 |  | 0.0267 | 0.0082 | 0.1328 | 0.1390 |
| *Torula* | 0.4670 | –0.1458 | –0.3913 | 0.0930 |  | 0.0070 | 0.4257 | 0.0268 | 0.6125 |
| *Trametes* | –0.2534 | 0.2042 | 0.2042 | –0.1760 |  | 0.1618 | 0.2622 | 0.2622 | 0.3354 |

**Supplementary Table 3.** Wilcoxon rank-sum test for fungal genera between busy and green sites in each season.

| Genera | Spring | |  | Genera | Summer | |  | Genera | Autumn | |  | Genera | Winter | |
| --- | --- | --- | --- | --- | --- | --- | --- | --- | --- | --- | --- | --- | --- | --- |
|  | *p*-value | corrected *p*-value |  |  | *p*-value | corrected *p*-value |  |  | *p*-value | corrected *p*-value |  |  | *p*-value | corrected *p*-value |
| *Alternaria* | 0.1939 | 0.7554 |  | *Alternaria* | 0.1832 | 0.5357 |  | *Cladosporium* | 0.5614 | 0.7436 |  | *Cladosporium* | 0.2338 | 0.6043 |
| *Cladosporium* | 0.7715 | 1 |  | *Penicillium* | 0.5491 | 0.5976 |  | *Alternaria* | 0.6631 | 0.7886 |  | *Alternaria* | 0.3719 | 0.6043 |
| *Phoma* | 1 | 1 |  | *Neurospora* | 0.3807 | 0.5357 |  | *Torula* | 1 | 1 |  | *Talaromyces* | 0.8817 | 0.9069 |
| *Talaromyces* | 1 | 1 |  | *Cladosporium* | 0.5516 | 0.5976 |  | *Talaromyces* | 0.3005 | 0.6648 |  | *Filobasidium* | 0.1215 | 0.6043 |
| *Didymella* | 0.877 | 1 |  | *Aspergillus* | 0.64 | 0.6656 |  | *Penicillium* | 1 | 1 |  | *Didymella* | 0.2186 | 0.6043 |
| *Naganishia* | 1 | 1 |  | *Naganishia* | 1 | 1 |  | *Aureobasidium* | 0.1215 | 0.6648 |  | *Fusarium* | 0.1859 | 0.6043 |
| *Fusarium* | 0.7568 | 1 |  | *Peyronellaea* | 0.4533 | 0.5357 |  | *Aspergillus* | 1 | 1 |  | *Penicillium* | 0.01771 | 0.6043 |
| *Penicillium* | 0.2784 | 0.7554 |  | *Neoascochyta* | 0.4533 | 0.5357 |  | *Neurospora* | 0.1859 | 0.6648 |  | *Phoma* | 0.64 | 0.72 |
| *Epicoccum* | 0.7389 | 1 |  | *Choanephora* | 0.4533 | 0.5357 |  | *Filobasidium* | 0.5271 | 0.7247 |  | *Torula* | 0.2059 | 0.6043 |
| *Microsphaeropsis* | 0.3529 | 0.7554 |  | *Didymella* | 0.4533 | 0.5357 |  | *Naganishia* | 0.1859 | 0.6648 |  | *Dothiorella* | 0.505 | 0.6269 |
| *Aspergillus* | 1 | 1 |  | *Fusarium* | 0.4533 | 0.5357 |  | *Epicoccum* | 0.7389 | 0.8555 |  | *Nothophoma* | 0.7389 | 0.8061 |
| *Exserohilum* | 0.7389 | 1 |  | *Trametes* | 0.4533 | 0.5357 |  | *Fusarium* | 0.2471 | 0.6648 |  | *Aureobasidium* | 0.505 | 0.6269 |
| *Scytalidium* | 1 | 1 |  | *Aureobasidium* | 0.4533 | 0.5357 |  | *Phoma* | 0.505 | 0.7168 |  | *Pseudogymnoascus* | 0.8676 | 0.9069 |
| *Aureobasidium* | 0.4533 | 0.7554 |  | *Cochliobolus* | 0.4533 | 0.5357 |  | *Stemphylium* | 0.8676 | 0.9789 |  | *Chaetomium* | 0.4533 | 0.6043 |
| *Coniothyrium* | 0.4533 | 0.7554 |  | *Coniothyrium* | 0.4533 | 0.5357 |  | *Curvularia* | 0.6084 | 0.7436 |  | *Coniothyrium* | 0.6084 | 0.7065 |
| *Filobasidium* | 0.4533 | 0.7554 |  | *Curvularia* | 0.4533 | 0.5357 |  | *Didymella* | 0.4533 | 0.6648 |  | *Dothidea* | 0.1859 | 0.6043 |
| *Nothophoma* | 0.4533 | 0.7554 |  | *Deniquelata* | 0.4533 | 0.5357 |  | *Dioszegia* | 0.6084 | 0.7436 |  | *Erythrobasidium* | 0.6084 | 0.7065 |
| *Paraconiothyrium* | 1 | 1 |  | *Kwoniella* | 0.4533 | 0.5357 |  | *Dothiorella* | 0.6084 | 0.7436 |  | *Papiliotrema* | 0.1859 | 0.6043 |
| *Phaeoacremonium* | 1 | 1 |  | *Leptosphaerulina* | 0.4533 | 0.5357 |  | *Coniothyrium* | 1 | 1 |  | *Arthrinium* | 0.4533 | 0.6043 |
| *Arthrinium* | 0.4533 | 0.7554 |  | *Moesziomyces* | 0.4533 | 0.5357 |  | *Metschnikowia* | 1 | 1 |  | *Aspergillus* | 0.4533 | 0.6043 |
| *Boeremia* | 0.4533 | 0.7554 |  | *Panaeolus* | 0.4533 | 0.5357 |  | *Nigrospora* | 0.1814 | 0.6648 |  | *Cystofilobasidium* | 1 | 1 |
| *Chaetomium* | 0.4533 | 0.7554 |  | *Pyrenochaetopsis* | 0.4533 | 0.5357 |  | *Seiridium* | 0.4533 | 0.6648 |  | *Epicoccum* | 0.4533 | 0.6043 |
| *Chaetopyrena* | 0.4533 | 0.7554 |  | *Saccharomyces* | 0.4533 | 0.5357 |  | *Acremonium* | 0.4533 | 0.6648 |  | *Occultifur* | 0.1814 | 0.6043 |
| *Dothiorella* | 0.4533 | 0.7554 |  | *Schizophyllum* | 0.4533 | 0.5357 |  | *Actinomucor* | 0.4533 | 0.6648 |  | *Acrocalymma* | 0.4533 | 0.6043 |
| *Paracamarosporium* | 0.4533 | 0.7554 |  | *Scopulariopsis* | 0.4533 | 0.5357 |  | *Alfaria* | 0.4533 | 0.6648 |  | *Kalmusia* | 0.4533 | 0.6043 |
| *Peniophora* | 0.4533 | 0.7554 |  | *Stemphylium* | 0.4533 | 0.5357 |  | *Arthrinium* | 0.4533 | 0.6648 |  | *Leptosphaerulina* | 0.4533 | 0.6043 |
| *Pseudogymnoascus* | 0.4533 | 0.7554 |  |  |  |  |  | *Beauveria* | 0.4533 | 0.6648 |  | *Microdochium* | 0.4533 | 0.6043 |
| *Rhodotorula* | 0.4533 | 0.7554 |  |  |  |  |  | *Clonostachys* | 0.4533 | 0.6648 |  | *Neodidymelliopsis* | 0.4533 | 0.6043 |
| *Setosphaeria* | 0.4533 | 0.7554 |  |  |  |  |  | *Cochliobolus* | 0.4533 | 0.6648 |  | *Neosetophoma* | 0.4533 | 0.6043 |
| *Torula* | 0.4533 | 0.7554 |  |  |  |  |  | *Coprinopsis* | 0.4533 | 0.6648 |  | *Neurospora* | 0.4533 | 0.6043 |
|  |  |  |  |  |  |  |  | *Coriolopsis* | 0.4533 | 0.6648 |  | *Nigrograna* | 0.4533 | 0.6043 |
|  |  |  |  |  |  |  |  | *Cyphellaceae* | 0.4533 | 0.6648 |  | *Nigrospora* | 0.4533 | 0.6043 |
|  |  |  |  |  |  |  |  | *Didymocyrtis* | 0.4533 | 0.6648 |  | *Paraboeremia* | 0.4533 | 0.6043 |
|  |  |  |  |  |  |  |  | *Lophotrichus* | 0.4533 | 0.6648 |  | *Sclerostagonospora* | 0.4533 | 0.6043 |
|  |  |  |  |  |  |  |  | *Myrmecridium* | 0.4533 | 0.6648 |  | *Sclerotinia* | 0.4533 | 0.6043 |
|  |  |  |  |  |  |  |  | *Nothophoma* | 0.4533 | 0.6648 |  | *Symmetrospora* | 0.4533 | 0.6043 |
|  |  |  |  |  |  |  |  | *Paraconiothyrium* | 0.4533 | 0.6648 |  |  |  |  |
|  |  |  |  |  |  |  |  | *Pyricularia* | 0.4533 | 0.6648 |  |  |  |  |
|  |  |  |  |  |  |  |  | *Rhodotorula* | 0.4533 | 0.6648 |  |  |  |  |
|  |  |  |  |  |  |  |  | *Sarocladium* | 0.4533 | 0.6648 |  |  |  |  |
|  |  |  |  |  |  |  |  | *Scytalidium* | 0.4533 | 0.6648 |  |  |  |  |
|  |  |  |  |  |  |  |  | *Setophoma* | 0.4533 | 0.6648 |  |  |  |  |
|  |  |  |  |  |  |  |  | *Sporobolomyces* | 0.4533 | 0.6648 |  |  |  |  |
|  |  |  |  |  |  |  |  | *Stachybotrys* | 0.4533 | 0.6648 |  |  |  |  |

**Supplementary Figure 1.** Fungal Strains isolated from each location (A) and month (B). Sampling district name (Nankai, Heping, Hexi, Hebei) and site (G = Green, B = Busy) are indicated.

**Supplementary Figure 2.** Strains of the dominant fungal genera isolated each month.

**Supplementary Figure 3.** Fungal genera isolated in the two environments (green vs busy) analyzed in each district.

**Supplementary Figure 4.** Strains isolated in the two analyzed environments (green vs busy).
